# Public Health Impacts of Water Fluorides: Current Evidence from a Rapid Systematic Review^☆^

**DOI:** 10.1016/j.advnut.2025.100547

**Published:** 2025-10-22

**Authors:** Fatemeh V Zohoori, Elizabeth A Kumah, Jelena Kronic, Michael Drinnan, Alexander J Morris

**Affiliations:** 1 School of Health and Life Sciences, Teesside University, Middlesbrough, United Kingdom; 2 Lloyd’s Register Foundation, London, United Kingdom; 3 School of Dentistry, University of Birmingham, Birmingham, United Kingdom

**Keywords:** fluoride, public health, human, community water fluoridation, nondental

## Abstract

Despite being established public health practice for >80 y, community water fluoridation continues to attract assertions of harm, and continued scrutiny of the evidence is required to inform policy. A rapid systematic literature review was conducted to examine human nondental health outcomes from fluoride exposure through drinking water. A protocol was developed a priori and registered on the Open Science Framework. Literature searches were conducted in Medline, CINAHL, Web of Science, Embase, PubMed, Campbell Collaboration, Cochrane Library, and Scopus databases. Gray literature and citation searches were also undertaken. Quality assessment was performed using the Mixed Methods Appraisal Tool (MMAT). Meta-analysis was judged not feasible due to the heterogeneous nature of the included studies. A total of 1143 unique records were identified, of which 130 full-text studies were reviewed and 58 were selected for final inclusion. Among these, 37 were cross-sectional studies, 15 were cohort studies, 4 were case-control studies, and 2 were ecological studies. Most studies were judged to be of acceptable quality using MMAT. The most common risk of bias was insufficient control of confounders. Health outcomes studied included blood pressure, neurological outcomes, bone cancers, thyroid function, skeletal outcomes, low birth weight/preterm birth, and metabolic outcomes. In children, some evidence of adverse effects on neurological outcomes and thyroid function was observed. In adults, some evidence was identified of adverse effects on blood pressure, thyroid function, and skeletal structure. In general, these effects occurred at drinking water fluoride concentrations >1.5 mg/L. No evidence of adverse effects was found at concentrations <1.0 mg/L in both children and adults. The nature of study designs and methodological limitations precluded causal inference. No convincing evidence of harm was identified from community water fluoridation at concentrations between 0.7 and 1.0 mg/L, whereas uncertainty remains at higher concentrations. Significant methodological limitations highlight the need for more rigorous future studies. A protocol was developed a priori and registered on the Open Science Framework (Registration DOI: https://doi.org/10.17605/OSF.IO/JH245).

**Statements of Significance**

This rapid systematic review provides the most up-to-date synthesis of post-2009 evidence on nondental human health outcomes from fluoride exposure via drinking water. It finds no convincing evidence of harm at recommended fluoridation concentrations (0.7 to 1.0 mg/L) and identifies key methodological limitations of existing studies that should guide future research and policy discussions.

## Introduction

According to the 2022 WHO’s Global Oral Health Status Report [1], almost 2 billion people suffer from dental caries of permanent teeth, whereas 514 million children experience caries of primary teeth [1]. Globally, untreated dental caries (tooth decay) in permanent teeth is the most prevalent health condition, and its prevalence continues to rise globally [1]. Oral diseases disproportionately affect the poor, with a strong link between socioeconomic status and the prevalence and severity of these conditions across all age groups and countries. Furthermore, treatments are expensive and often excluded from health coverage [1].

Oral health is essential to overall health and quality of life. Dental caries, pain, and treatment can have a profound negative impact on general health and well-being, leading to pain, infection, and difficulties with eating and sleeping, as well as affecting self-esteem and psychological health [2]. Furthermore, poor oral health has been associated with increased risk of various conditions, including pneumonia, cardiovascular disease, stroke, low birth weight, and endocarditis [2].

Oral diseases impose a substantial global economic burden, with estimated annual costs of US$387 billion in direct expenditures and US$323 billion in productivity losses. Direct costs primarily arise from public and private spending on dental care, whereas indirect costs reflect reduced productivity. Among indirect costs, edentulism accounts for the largest share (US$167 billion). Dental caries contributes additional indirect costs of ∼US$22 billion for permanent teeth and US$ 1.55 billion for deciduous teeth [3].

Apart from good oral hygiene and a balanced diet low in sugar consumption, fluoride has been widely recognized for its ability to reduce the prevalence and severity of dental caries [4]. Fluoride is a naturally occurring mineral found in soil, food, and water supplies, with concentrations varying by region. Due to its role in the mineralization of hard tissues, including bones and teeth, fluoride can have a significant public health impact in reducing the burden of dental caries [5]. This protective effect is primarily topical, as fluoride enhances remineralization, inhibits demineralization of tooth enamel, and disrupts bacterial metabolism, reducing acid production [5].

Although fluoride at low concentrations is well documented for preventing dental caries, higher concentrations of systemic fluoride exposure may result in the development of dental fluorosis. To optimize oral health outcomes while mitigating risks of dental fluorosis (the most sensitive effect), the WHO recommends a maximum fluoride concentration of 1.5 mg/L in drinking water [6].

To lower the prevalence of dental caries, fluoride has been incorporated into various public health initiatives, such as water, salt, and milk fluoridation programs, alongside its addition to dental products like fluoridated toothpaste, mouthrinses, and professionally applied varnishes and gels. Among public health fluoridation programs, water fluoridation remains the most widely practiced intervention to reduce the prevalence and severity of dental caries and address dental health inequalities. Water fluoridation programs serve ∼400 million people across 25 countries [7], including the United States, Australia, New Zealand, Brazil, the United Kingdom, and Ireland.

A substantial body of evidence supports the benefits of water fluoridation for both children and adults, including the prevention of dental caries and reduction of oral health inequalities. Systematic reviews [[8], [9], [10]] have demonstrated that community water fluoridation reduces the incidence of dental caries by 26 to 44% in both children and adults, providing benefits across all age groups and socioeconomic strata, regardless of access to dental care. Despite the widespread use of fluoride toothpaste, water fluoridation offers a complementary and additive effect: whereas fluoride toothpaste delivers topical protection during brushing, fluoridated water ensures consistent low-concentration fluoride exposure throughout the day, supporting continuous remineralization of early carious lesions [11,12].

Despite its proven benefits, there is ongoing debate about the potential harmful effects of systemic fluoride exposure on human health. Although extensive research highlights both the dental benefits of fluoride and risks associated with high concentrations of fluoride intake, the broader nondental health implications of water fluoridation remain poorly understood. In some parts of the world, drinking water naturally contains fluoride concentrations greatly in excess of WHO recommendations, and this may increase risk of skeletal fluorosis [6]. Despite continued support from the United States Centers for Disease Control and Prevention [13] 2 states in the United States have recently passed legislation to ban water fluoridation, citing health concerns [14].

To address this gap, our rapid review aimed to systematically explore the existing literature on the nondental human health impacts of water fluoridation. Unlike previous studies, which predominantly rely on narrative literature review methods, this review adopts a systematic review methodology to synthesize evidence on the effects of water fluoridation on human health. In response to recent global debates on fluoride safety and use, a rapid systematic review was necessary to provide timely, robust, reliable, and valid evidence. Rapid reviews adhere to the core principles of systematic review, such as having clearly defined research questions and a transparent and replicable methodology, while streamlining certain steps, including limiting the publication date of eligible studies and reducing the number of reviewers involved at each stage of the review process, to deliver results more quickly. This approach balances methodological rigor with practical feasibility, making it particularly suitable for fast-moving policy environments where timely guidance is essential [15].

## Research Question

This rapid systematic review aimed to answer the following question:

• What is the nondental human health impact of water fluoridation?

## Methods

The Preferred Reporting Items for Systematic Reviews and Meta-Analysis (PRISMA) checklist [16] was used to guide the conduct and reporting of this systematic review. A protocol was developed a priori and registered on the Open Science Framework (Registration DOI: https://doi.org/10.17605/OSF.IO/JH245).

### Search strategy and reference management

An experienced evidence synthesis specialist (EAK) developed the search strategy, which was subsequently reviewed by a public health expert with extensive expertise in fluoride research (FVZ). The search incorporated a combination of key concepts and terms aligned with the research question. These included phrases such as “water fluoride intake,” “water fluoride exposure,” “public health impact,” “health impact,” and “human health impact.”

A comprehensive search was conducted across multiple sources, including online databases (Medline, CINAHL, Web of Science, Embase, PubMed, Campbell Collaboration, Cochrane Library, and Scopus), search engines (e.g., Google Scholar), and gray literature repositories (such as NICE Evidence Search and the Gray Literature Report). In addition, reference lists of eligible studies were manually screened to identify any further relevant publications. The search was carried out on January 24 and January 27, 2025, and was limited to studies published from January 2010 onward. Due to language translation limitations, only studies published in English were included. A detailed example of the search strategy used for the CINAHL database (via EBSCOhost) is provided in Supplementary File A.

Search results were initially imported into an EndNote library for reference management and to facilitate the removal of duplicate records. The deduplicated search results were then transferred to Covidence (Covidence.org), a systematic review management platform, for title and abstract screening (an initial assessment of studies against prespecified eligibility criteria to identify potentially relevant articles), full-text review, quality assessment, and data extraction. Covidence also assisted in identifying and removing any remaining duplicates before commencing literature screening.

#### Study selection

Titles and abstracts were double-screened by 2 independent reviewers (EAK and FVZ) for 20% of the search results, with the remainder screened by 1 reviewer (EAK or FVZ). Disagreements between reviewers were resolved through discussion. Full-text screening was undertaken by 1 reviewer (EAK or FVZ).

#### Selection criteria

Eligible studies were selected using the inclusion and exclusion criteria presented in Table 1.

**TABLE 1:** Inclusion and exclusion criteria for study selection.

	Included	Excluded
Population	All humans of any age.	Animals
Settings	All countries and settings with naturally occurring water fluoride or adjusted water fluoridation.	Settings where there is no adjusted water fluoridation or naturally occurring fluoride in water supplies.
Context	This included, but not limited to, schools, early childhood care centers, community centers, clinics, and hospitals.	
Intervention/ exposure	Water fluorides of any concentration	All other exposures
Outcomes	Nondental human health outcomes including, but not limited to, hip fractures, renal calculi, down syndrome, bladder cancer, and osteosarcoma. We also included specific indicators as reported in eligible studies, looking at, for example, the effect of water fluoride on IQ and cognitive development.	Nonclinical or nonhealth outcomes, dental outcomes
Language	Studies with full texts in the English language.	Studies with full texts in other languages
Date of publication	Primary studies published from January 2010 to date.	Studies published before January 2010
Study design	Experimental studies (Randomized Controlled Trials, Quasi-Experimental Designs), observational studies (cohorts, case-control, and cross-sectional studies, as well as case reports and case series) and ecological studies.	Systematic or narrative reviews (with or without meta-analysis), guidelines, modeling studies, laboratory studies.
Publication type	Peer-reviewed studies and preprints	Non-peer-reviewed sources such as editorials, commentaries, opinion pieces, letters without original data, conference abstracts without full-text data

### Data extraction

A standardized data extraction form was developed within the Covidence software to guide the data extraction process. Extracted information included study title, aims/objectives/research questions, country of study, study design, participant demographics, setting, assessed outcomes, outcome measures, fluoride concentrations, results, and relevant contextual information. The form was pilot tested on 20% of the included studies to ensure consistency and clarity before full data extraction commenced. Data extraction was carried out by 1 reviewer (JK) and independently checked for accuracy and completeness by a second reviewer (EAK).

#### Assessment of methodological quality

The quality assessment of all included studies was conducted using the Mixed Methods Appraisal Tool (MMAT) [17]. One reviewer (JK) performed quality assessment, which was independently verified by a second reviewer (EAK). The MMAT is a validated critical appraisal tool designed to assess the methodological quality of a wide range of study designs, including randomized controlled trials, nonrandomized studies, qualitative research, quantitative descriptive studies, and mixed-methods studies. Any discrepancies between reviewers were resolved through discussion to reach consensus.

#### Data synthesis

A meta-analysis was initially planned, contingent upon sufficient homogeneity in water fluoride concentration, participant characteristics, and outcome measures across the included studies. However, substantial heterogeneity in these key domains rendered a quantitative synthesis inappropriate. Consequently, the findings were summarized descriptively.

## Results

### Search results

**FIGURE 1:**
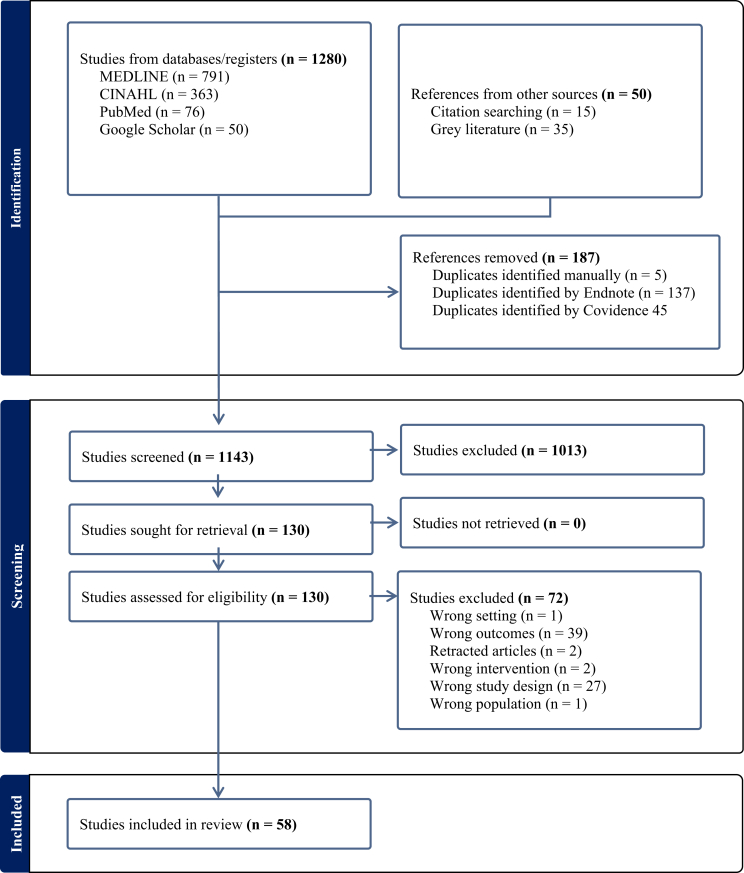
PRISMA flow diagram; n = number.

The study selection process is illustrated in Figure 1. The database and search engine searches yielded a total of 1280 records, with an additional 50 records identified through gray literature and citation searching. After the removal of duplicates, 1143 unique records remained. A total of 1013 records were excluded during title and abstract screening, leaving 130 articles for full-text review. Of these, 72 were excluded for various reasons (including wrong setting, outcomes, interventions, study design, and/or study population), resulting in 58 studies being included in the final systematic review (Figure 1).

### Study characteristics

**FIGURE 2:**
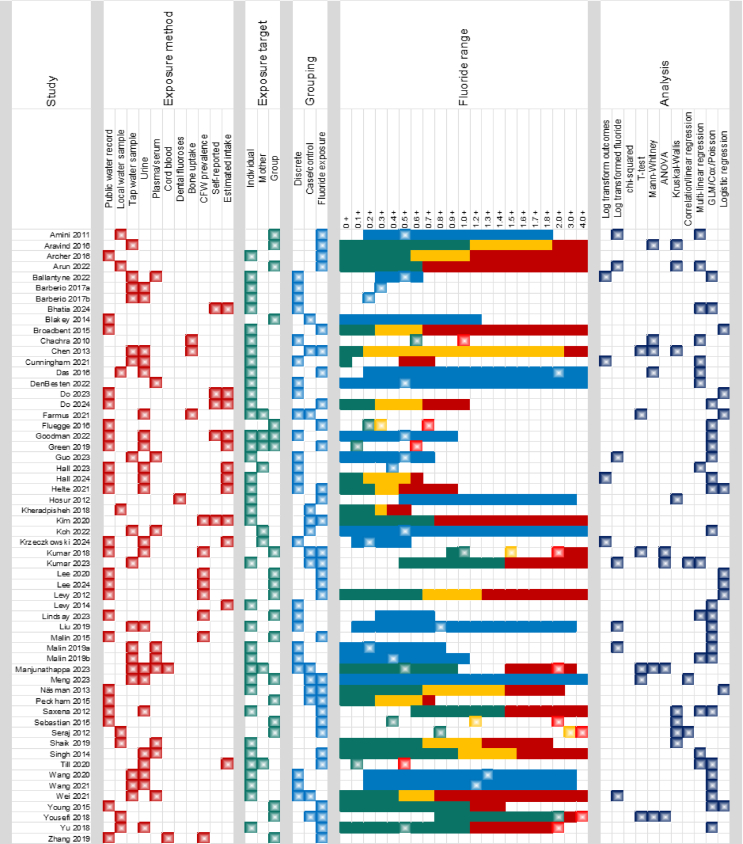
Comparative overview of fluoride exposure assessment methods, exposure grouping, concentration ranges, and analytical approaches across studies. Fluoride range: Green, amber, and red bars represent the fluoride concentration ranges for authors’ definitions of low-, medium-, and high-exposure groups, respectively. Blue bars indicate the overall fluoride range in studies where group classifications were not reported or not applicable. Stars mark the central value (mean or median) of the reported fluoride concentration, where available.

A summary of the study characteristics is presented in Table 2 and Figure 2, with detailed information provided in Supplementary File B.

**TABLE 2:** Summary of characteristics of included studies.

Classification	Frequency Number (%)
Study design	
Cross-sectional	37 (63.8)
Cohort study	15 (25.9)
Case-control study	4 (6.9)
Ecological study	2 (3.4)
Year of publication	
2010–2013	8 (13.6)
From 2014	50 (86.4)
Water fluoride concentration^1^	
Low (≤ 0.7 mg F/L)	35 (60.3)
Optimal (< 1.5 mg F/L)	25 (43.1)
High (≥ 1.5 mg F/L)	18 (31.0)
Types of participants	
Children (aged 0–12)	15 (22.5)
Adolescents (aged 13–17)	1 (1.7)
Adults (aged ≥ 18)	14 (24.1)
Children and adolescents	8 (13.8)
Children, adolescents, and adults	20 (37.8)

1 Some studies involved participants exposed to varied concentrations of water fluoride concentration and were therefore counted in multiple categories, as classified by the original study authors.

Findings from this systematic review indicate that most studies assessing the impact of water fluoride concentration on human health were conducted in the United States (n = 17), followed by Canada (n = 11), India (n = 10), China (n = 6), Iran (n = 4), and the United Kingdom (n = 3). Two studies each were conducted in Australia, South Korea, and Sweden, whereas 1 study originated from New Zealand. In terms of study design, most studies employed a cross-sectional approach (n = 37), followed by cohort studies (n = 15), case-control studies (n = 4), and ecological studies (n = 2). The included studies were published between 2010 and 2024, with the majority (n = 50) published from 2014 onward. Regarding the study populations, 14 studies focused exclusively on adults aged ≥18 y. Three studies involved children aged 0 to 5 y, and 7 involved children aged 6 to 12 y. One study focused solely on adolescents aged 13 to 17 y. A total of 33 studies included participants of varied ages, with populations comprising children (0–12 y), adolescents, and adults in different combinations. Participants were exposed to a range of water fluoride concentrations, from 0.01 mg F/L to 10.30 mg F/L (Table 2).

Figure 2 presents a comparative overview of studies investigating fluoride exposure, highlighting key differences in measurement methods, exposure classification, concentration ranges, and statistical analyses. Fluoride exposure was assessed using diverse methodologies, including direct measurement from water sources, biomonitoring, and modeled estimates. Several studies categorized fluoride exposure into discrete groups (e.g., low, medium, high), whereas others treated it as a continuous variable. Reported exposure ranges varied considerably, from 0.01 mg F/L to 10.30 mg F/L, with some studies examining narrow exposure bands and others capturing a broader distribution. Measures of central tendency, such as the mean or median, were reported inconsistently across studies. Statistical approaches ranged from basic bivariate analyses to more complex multivariable models, reflecting heterogeneity in methodological rigor and analytical depth across the literature.

### Assessment of methodological quality

To assess the methodological quality of the included studies, criteria from the MMAT were applied, specifically those relevant to nonrandomized controlled studies (including cross-sectional, cohort, and case-control designs) and quantitative descriptive studies (including ecological studies). Fifty-seven studies were appraised using the 5 criteria items under the nonrandomized controlled studies domain, whereas 1 ecological study was assessed using the 5 criteria under the quantitative descriptive domain.

**FIGURE 3:**
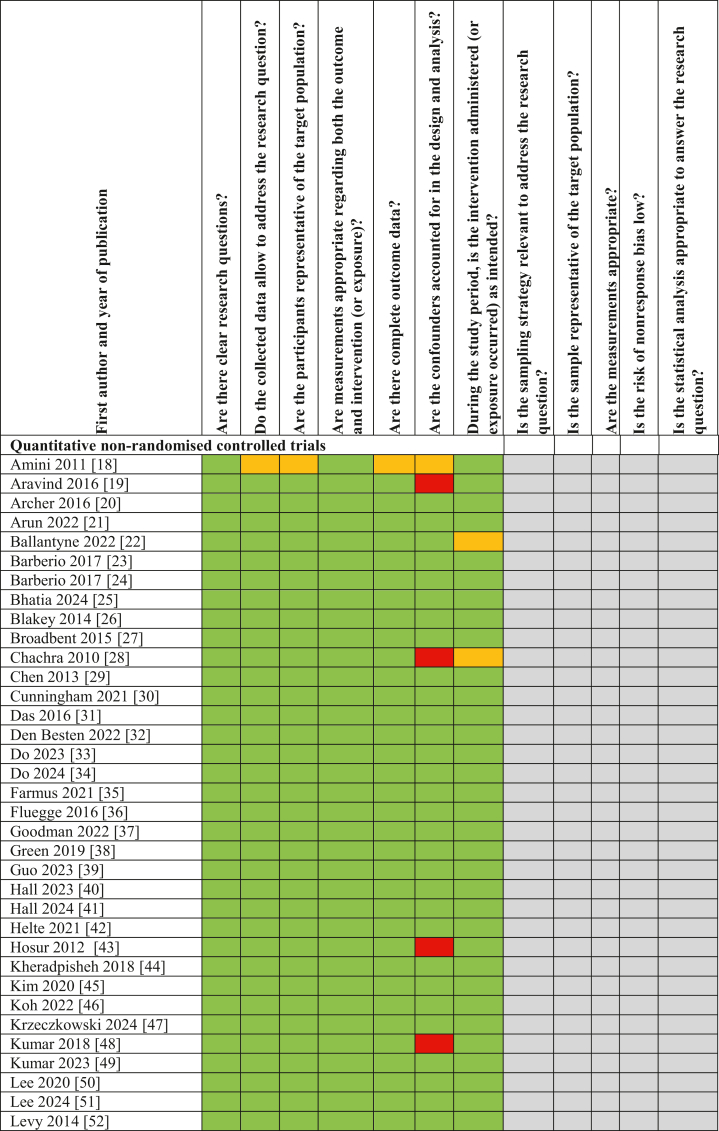

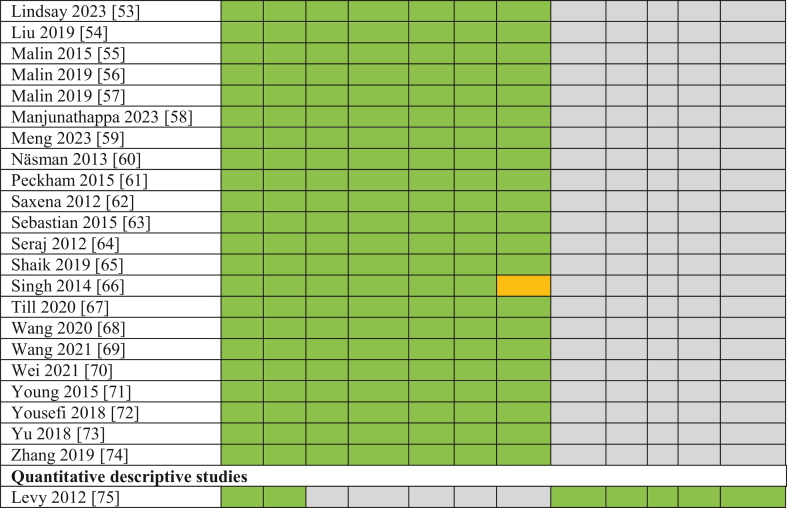
Methodological quality scores for included studies using the Mixed Methods Appraisal Tool. Color codes: Green – Yes, Red – No, Yellow – Unclear, Gray – Not applicable [[90], [91], [92], [93], [94], [95]].

Prior to the full quality assessment, the 2 MMAT prescreening questions were applied to each study to confirm eligibility for further appraisal. The detailed quality assessment outcomes, including the MMAT criteria applied and individual study scores, are presented in Figure 3. All included studies met ≥1 of the prescreening criteria. Of the 58 studies assessed, 52 met all 5 MMAT quality criteria, and 4 met 4 out of 5. Overall, the majority of studies were assessed to be of “very good” methodological quality. Among the few studies that did not meet all criteria, the most common limitation was insufficient consideration or control of confounding variables in the study design and analysis.

### Impacts of water fluorides on nondental human health

#### Cardiovascular outcomes (systolic and diastolic blood pressure)

In children, 3 studies [[18], [19], [20]] found no effect of fluoride exposure on blood pressure. In adults, however, 2 studies [21,22] conducted in areas with fluoride concentrations ranging from 0.23 to 10.30 mg/L reported higher blood pressure associated with higher concentrations of fluoride exposure.

#### Obesity outcomes (BMI and waist circumference)

In children, findings were inconsistent: 1 study reported a positive association between higher fluoride exposure and increased BMI (in kg/m^2^) [23], whereas another observed an inverse relationship [24]. In adults, evidence was limited, with 1 study finding no significant association between fluoride exposure and BMI [22].

#### Neurological outcomes IQ and neurocognitive development)

In children, many studies [[25], [26], [27], [28]] have found no significant association between fluoride exposure and neurodevelopmental outcomes at concentrations of 1 mg/L, though some research suggests potential neurotoxic effects at this concentration [[29], [30], [31]]. More pronounced effects, such as lower IQ scores and impaired neurocognitive function, have been reported at fluoride concentrations exceeding 1.5 mg/L [24,[32], [33], [34], [35], [36], [37], [38], [39], [40]]. In adults, the evidence remains limited and inconclusive regarding any neurotoxic effects of fluoride exposure.

#### Cancer outcomes (osteosarcoma, Ewing sarcoma)

In both children and adults [[41], [42], [43], [44], [45], [46]], studies have found no clear evidence of an association between fluoride exposure, including at concentrations >2 mg/L, and risk of osteosarcoma or Ewing Sarcoma.

#### Thyroid function outcomes (hyperthyroidism, TSH, T3, T4 hormones, hyperuricemia)

In both children [39,[47], [48], [49], [50]] and adults [28,48,51,52], some evidence suggests that fluoride exposure, particularly at concentrations >1.5 mg/L, may be associated with alterations in thyroid function, including changes in TSH, T3, and T4 concentrations. However, other studies have found no significant effects of fluoride on thyroid function [47,[53], [54], [55]].

#### Skeletal outcomes (lumbar and femoral bone mineral density, osteoporosis, osteoarthritis, hip/arm/other fractures)

In adults, overall evidence suggests that low-concentration fluoride in drinking water has no significant harmful effect on bone health in the general population, likely due to multiple other contributing factors [44,45,56,57]. A weak association has been observed between fluoride exposure, accumulated fluoride, and the physical characteristics of bone, with the high variability in heterogeneous urban populations likely masking any subtle effects of low-concentration fluoride on skeletal tissue [58]. However, in postmenopausal women, fluoride exposure at concentrations ≥1 mg/L has been associated with increased bone mineral density but also a higher risk of fractures [59,60].

In children, fluoridated tap water at ∼0.8 mg/L was linked to a decreased risk of bone fractures [44] suggesting some protective effects. Additionally, community water fluoridation programs have not been shown to increase risks of bone diseases [44,45,61].

#### Birth outcomes (low birth weight, preterm birth)

Findings on birth outcomes were inconsistent across studies. Two studies reported no significant associations: 1 [62] found no link between community water fluoridation (0.7–1.0 mg/L) and preterm birth, whereas the other [63] observed no associations between maternal fluoride exposure and outcomes such as fertility, birth weight, gestational age, preterm birth, or small-for-gestational-age. However, in contrast, 1 study [64] reported a significant association between water fluoride concentrations >0.7 ppm and low birth weight among Hispanic women.

#### Metabolic outcomes (vitamin D deficiency, fasting blood glucose, diabetes, sleep disorder)

Vitamin D deficiency and elevated blood glucose concentrations were observed in patients residing in fluoride-endemic areas (fluoride concentration >1.5 mg/L), whereas both parameters were within normal ranges in individuals from areas with fluoride concentrations ≤1.5 mg/L [65].

Community water fluoridation has been linked to diabetes-related epidemiological outcomes, with adjusted fluoridated water (≤1.8 mg/L) associated with increased incidence and prevalence, whereas naturally fluoridated water (≤1.1 mg/L) appears to have a protective association. [66].

Fluoride exposure at concentrations ≤ 0.8 mg/L has been linked to shorter sleep duration among individuals aged 16 to 79 y [67]. However, a separate study focusing on adolescents aged 16 to 19 y found no significant association between fluoride exposure and sleep duration, although participants reported symptoms indicative of sleep apnea [68].

## Discussion

Recent global discussions on the use of fluoride for preventing dental caries have emphasized the need for timely, evidence-informed public health guidance. Using a rapid review approach, this study generated a structured overview of current evidence on the nondental health impacts of water fluoridation, balancing comprehensiveness with practical feasibility. This review systematically assessed multiple health domains to clarify potential risks and knowledge gaps. The findings indicate that community water fluoridation at concentrations ≤ 1.0 mg/L is not associated with adverse nondental health outcomes, whereas the evidence for concentrations between 1.0 and 1.5 mg/L is limited and less conclusive.

Despite 8 decades of public health implementation of water fluoridation, particularly at concentrations between 0.7 and 1.0 mg/L, the literature on its broader human health impacts remains fragmented and heterogeneous. The studies reviewed in this article investigated populations exposed to fluoride concentrations in drinking water ranging from 0.01 mg/L to 10.30 mg/L. This review focused on 8 key nondental health domains: cardiovascular outcomes, obesity, neurological development, cancer, thyroid function, skeletal health, birth outcomes, and metabolism. However, drawing robust conclusions within each of these domains is challenging, given the variability in study populations and settings. Studies were conducted across diverse countries, including the United States, Canada, China, India, Australia, South Korea, Sweden, and New Zealand, and in a variety of contexts such as homes, schools, communities, clinics, hospitals, and population databases. Additionally, there was significant variation in study designs (cross-sectional, case-control, cohort, interventional, and ecological) and methods for measuring fluoride exposure (public water records, groundwater samples, etc.). Moreover, inconsistencies in age groups and outcome measurement methods further complicate comparisons, making it difficult to establish definitive conclusions.

Across the 58 studies included in this rapid systematic review, the evidence largely supports the safety of current fluoridation concentrations (0.7–1.0 mg/L) commonly used in public health programs. However, evidence of potential adverse health effects becomes more pronounced at fluoride concentrations above those typically used in community water fluoridation (i.e., >1.0 mg/L). To facilitate dose–response interpretation, studies were stratified based on fluoride concentrations in drinking water: low/acceptable exposure (≤1.5 mg/L), in line with the WHO guideline, and high exposure (>1.5 mg/L).

Thyroid-related outcomes were among the most frequently examined. Several studies reported statistically significant associations between high fluoride exposure and alterations in thyroid hormone regulation [28,39,[47], [48], [49], [50], [51], [52]]. Elevated TSH concentrations and altered T3 and T4 concentrations were observed in both children and adults [47,49,51,52]. However, other studies [49,55,69] found no associations, particularly in populations with adequate iodine intake. In general, none of these studies accounted for factors that influence thyroid function, which is strongly affected by nutrition and the gut–thyroid axis. Micronutrients such as iodine, selenium, iron, zinc, copper, magnesium, vitamin A, and vitamin B12 play key roles in hormone synthesis and regulation, and dietary imbalances can alter gut microbiota, impair nutrient absorption and immune regulation, and contribute to thyroid dysfunction [70].

Findings related to blood pressure and cardiovascular health were mixed. Although some studies observed possible links between higher fluoride exposure and increased hypertension prevalence [21, 22], others found no clear effects [18,20], or even inverse associations in younger populations [19]. However, none of these studies accounted for key confounding factors such as obesity, diabetes, smoking, and dietary habits, which are well-established risk factors for cardiovascular disease and hypertension [71].

Overall, evidence indicates that fluoride concentrations typical of community water fluoridation do not significantly harm bone health in the general adult population. However, in postmenopausal women, fluoride exposure at concentrations ≥1 mg/L has been associated with increased bone mineral density as well as a higher risk of fractures [59,60]. In children, fluoridated water at ∼0.8 mg/L appears protective, correlating with a reduced risk of bone fracture [44].

Evidence concerning fluoride’s carcinogenic potential, particularly in relation to osteosarcoma, predominantly indicates no significant association between fluoride exposure and cancer incidence even at elevated concentrations [[41], [42], [43], [44], [45], [46]]. This finding is consistent with a recent systematic review [72] on fluoride exposure and risk of primary bone cancer, which reported no clear association in 12 out of 14 included studies. The 2 studies that did report a positive association between fluoride exposure and bone cancer in young males were published in 1992 [73] and 2001 [74], dates that fall outside the inclusion period for this rapid review.

Evidence for fluoride’s impact on birth outcomes was limited and inconsistent. Some studies reported associations between higher fluoride exposure and increased risk of low birth weight or preterm birth [64], whereas others found no significant relationships [62,63].

Findings related to metabolic health were sparse and heterogeneous. Conditions such as vitamin D deficiency, elevated blood glucose concentrations, and diabetes have been observed in some studies conducted in fluoride-endemic areas (fluoride concentration >1.5 mg/L) [65,66]. However, these conditions are also common in the general population and are influenced by many factors (such as inadequate sun exposure, poor diet, limited physical activity, socioeconomic disadvantage, genetics, and obesity [75]) that are unrelated to fluoride exposure. Other investigations have not found consistent associations between fluoride exposure and outcomes such as BMI or general metabolic dysfunction [23,24].

Findings in the neurodevelopmental domain included reports that prenatal maternal and early childhood fluoride exposure may be associated with cognitive development in young children [29, 31,37,39,40]. However, these findings have faced methodological criticism [76,77], and other studies have not replicated this association, particularly in settings with lower exposure concentrations and more robust adjustment for confounding factors [25,27]. A meta-analysis of 8 observational studies from nonendemic fluorosis areas found no statistically significant association between fluoride concentrations typical of community water fluoridation and children's IQ scores. [78]. Similarly, recent reviews [[79], [80], [81]] on the effects of fluoride on cognitive neurodevelopment have reported possible IQ impairment only at exposures exceeding WHO guidelines, with no evidence of effects at lower concentrations (<1.5 mg/L). However, high heterogeneity across studies limits the validity of these findings. However, a recent systematic review and meta-analysis of 74 studies [82] reported that when fluoride was measured in water, the negative association with children's IQ scores was significant only at higher concentrations (>2 mg/L) and not <1.5 mg/L. In contrast, when fluoride was measured in urine, the inverse association remained significant even at concentrations < 1.5 mg/L. Notably, nearly all studies included in this meta-analysis that used urinary fluoride as an exposure indicator relied on spot urine samples. Twenty-four-hour urinary fluoride excretion has been suggested as a valid and reliable indicator of short-term fluoride exposure. Since the concentration of analytes, such as fluoride, can fluctuate throughout the day, spot urine samples may not accurately represent total 24-h urinary excretion and, therefore, may not provide a true estimate of fluoride exposure. Furthermore, a recent study [83] reported significant differences in estimated fluoride intake and in the classification of children into intake categories (such as low, intermediate, and high exposure) when comparing urine-based estimates with those obtained from dietary and oral hygiene questionnaires. These findings underscore the limitations of relying solely on spot urine samples for assessing fluoride exposure.

Overall, this rapid review suggests that age, fluoride dose, and exposure context may influence potential health outcomes, though the evidence is mixed and methodologically heterogeneous. Some studies point to greater susceptibility in children, particularly regarding neurological and skeletal effects, whereas in adults, associations are more often reported in thyroid and skeletal domains. However, inconsistent findings, reliance on cross-sectional designs, variable exposure assessment methods, and potential risks of bias (such as confounding and selection bias) limit the validity of the evidence and the strength of the conclusions.

Although fluoride concentrations used in community water fluoridation (0.7–1.0 mg/L) are generally considered safe, some studies found statistically significant associations at concentrations above the WHO guideline (1.5 mg/L). The clinical significance of these findings remains uncertain, and potential risks may depend on total fluoride intake, pre-existing health conditions, and individual-level factors such as metabolic rate and nutritional status. Further high-quality longitudinal research is needed to clarify these relationships.

An understanding of fluoride’s pharmacokinetics and primary exposure pathways is essential for interpreting its health impact, given its biphasic nature—being beneficial at low concentrations but potentially causing adverse effects with excessive systemic exposure.

Fluoride is naturally present in trace amounts in the human body (∼2.6 g) [12]. Although the dental health effects of fluoride are primarily topical, the nondental effects result from systemic ingestion. Systemic fluoride exposure mainly occurs through the consumption of fluoridated water, and in children, through unintentional ingestion of fluoride-containing dental products. Once ingested, fluoride is rapidly absorbed through the gastrointestinal tract, reaching peak plasma concentrations within 20 to 60 min [84]. Approximately 99% of the fluoride present in the body is found in calcified tissue like bones and teeth, with the kidneys responsible for most of its elimination [84]. Retention, however, varies by age, with about 35% retained in adults and ≥ 55% in children [85], reflecting differences in metabolism and skeletal growth. Several factors influence fluoride metabolism and modify the relationships among its intake, bodily retention, and associated health risks [84]. These factors include genetics, diet composition, nutritional status, physical activity, renal function, and acid-base balance [84].

Many studies estimate fluoride exposure based on community water concentrations; however, this does not fully capture individual intake from other sources. The primary contributors to systemic fluoride exposure are diet and inadvertent ingestion of fluoride-containing dental products. On average, toothpaste contributes ∼6% of total fluoride intake in infants <12 mo of age and ∼22% in children aged 1 to 4 y [86]. However, in children < 4 y, unintentional ingestion of toothpaste can account for ≤ 87% of total daily fluoride intake, depending on age, toothpaste quantity, and rinsing behavior [87]. In contrast, infants < 12 mo primarily receive fluoride through their diet, which includes fluoridated water, dietary fluoride supplements (such as fluoridated water, milk, and salt), and foods and beverages prepared with fluoridated water.

In some studies, urinary fluoride has been employed as a biomarker of fluoride exposure to evaluate its potential health effects. Although it is a reliable indicator of recent fluoride intake [85], urinary fluoride concentrations are influenced by various factors, including dietary patterns (e.g., vegetarian versus meat-based diets), altitude, hydration status, and renal function [84]. The predominance of cross-sectional study designs in the current literature constrains the ability to infer causality. Furthermore, methodological heterogeneity, along with variability in population demographics and regional risk profiles, contributes to inconsistent associations observed across health outcomes.

Across all nondental health impact domains examined in this rapid review, a key limitation of the included studies was the insufficient consideration of a comprehensive range of confounding factors. For instance, iodine deficiency, a leading cause of reduced IQ and impaired cognitive development, was rarely accounted for, despite its significant impact on neurological outcomes. Additionally, other environmental factors such as socioeconomic status, nutritional deficiencies, and coexposure to multiple pollutants (e.g., lead) were often overlooked. This lack of thorough confounder adjustment undermines the ability to isolate the possible effects of fluoride exposure on health outcomes and may contribute to the inconsistent findings observed in the literature.

## Conclusion

This rapid systematic review indicates that community water fluoridation at concentrations between 0.7 and 1.0 mg/L is not associated with adverse nondental health effects in the general population. However, exposures exceeding the WHO guideline of 1.5 mg/L may increase risk of health effects, particularly among children and other susceptible groups. The review highlights the importance of considering total fluoride intake from multiple sources, age-related susceptibility, and individual factors such as metabolism and nutritional status. Given the heterogeneity of existing studies and inadequate adjustment for confounders, future research should comprehensively investigate potential health effects across all exposure concentrations, with particular focus on rigorous longitudinal studies with valid measurement of exposure that address these gaps.

### Policy implications

The evidence reviewed in this study reinforces the continued endorsement of community water fluoridation at concentrations between 0.7 and 1.0 mg/L as a safe and effective public health intervention for the prevention of dental caries. Community water fluoridation remains one of the most equitable and cost-effective strategies for reducing oral health disparities, particularly among children and low-income populations. A recent cost-effectiveness analysis based on data from 8484 children (mean age 9.6 y) from the 2013–2016 United States NHANES [88] estimated that the elimination of fluoridation would result in a 7.5 percentage point increase in dental caries and an associated $9.8 billion in additional treatment costs over 5 y. In addition to this substantial economic burden, community water fluoridation is estimated to save ∼$20 in dental treatment costs for every $1 invested, demonstrating strong cost-effectiveness [89]. This underscores the considerable cost-savings and the well documented clinical consequences, such as increased dental decay and related oral health complications, that would arise from withdrawing community water fluoridation.

To safeguard public health benefits while minimizing potential risks, regulatory agencies should prioritize regular monitoring of fluoride concentrations in community water supplies to ensure adherence to recommended concentrations and prevent overexposure. Policy frameworks should also account for cumulative fluoride intake from multiple sources, including dental products, dietary supplements, and regionally variable environmental contributions.

Special consideration should be given to vulnerable groups, such as infants, young children, and individuals with nutritional or metabolic susceptibilities. In these populations, targeted risk assessment and tailored preventive strategies may be warranted.

Finally, effective risk communication is essential. Public health authorities must provide transparent, evidence-based messaging regarding the benefits and potential risks of fluoride exposure in order to maintain public trust and support informed decision-making.

### Future research recommendations

Future studies should prioritize well-designed longitudinal cohort studies with comprehensive fluoride intake assessment, incorporating all significant sources of intake beyond drinking water, including dental products and diet. It is critical to adjust for a broad spectrum of confounding variables, such as iodine status, socioeconomic factors, nutritional deficiencies, and coexposures to environmental pollutants, to clarify any causal relationships between fluoride exposure and health outcomes.

More research is needed to elucidate fluoride’s dose–response effects across different age groups, particularly children <12 y, who appear more vulnerable to neurological impacts. Investigations into the pharmacokinetics of fluoride in diverse populations, including genetic and metabolic variations, will further inform safe exposure thresholds. Finally, harmonizing outcome measures and fluoride exposure metrics across studies will enhance comparability and strengthen evidence synthesis.

## Author contributions

The authors’ responsibilities were as follows – FVZ and EAK conceived and designed the study. FVZ and EAK conducted the screening of study titles, abstracts, and full texts, and assessed eligibility. JK and EAK performed data extraction and quality assessment. MD, FVZ, and EAK conducted the data synthesis. FVZ and EAK drafted the initial manuscript. AJM and MD critically reviewed the manuscript and provided intellectual feedback. FVZ had primary responsibility for the final content. All authors read and approved the final manuscript.

## Data availability

All data underlying the results of this systematic review are available in the article and its supplementary materials. No new primary data were collected**.**

## Funding

The authors reported no funding received for this study.

## Conflict of interest

The authors report no conflicts of interest.
