# Supplementary material for: Public Health Impacts of Water Fluorides: Current Evidence from a Rapid Systematic Review
Source: Adv Nutr. 2025 Oct 22;16(12):100547. doi: 10.1016/j.advnut.2025.100547 (PMC12662105; doi:10.1016/j.advnut.2025.100547)
Supplement: multimedia component 1 [file mmc1.docx]

**File A: Detailed example of the search strategy used for the CINAHL database**


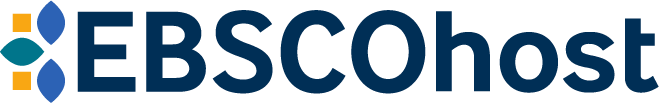


| **#** | **Query** | **Limiters/Expanders** | **Last Run Via** | **Results** |
| --- | --- | --- | --- | --- |
| S37 | S10 AND S16 AND S35 | Limiters - Publication  Date: 20100101-  20251231  Expanders - Apply equivalent subjects Search modes - Proximity | Interface - EBSCOhost  Research Databases  Search Screen - Advanced  Search  Database - CINAHL Complete | 792 |
| S36 | S10 AND S16 AND S35 | Expanders - Apply equivalent subjects Search modes - Proximity | Interface - EBSCOhost  Research Databases  Search Screen - Advanced  Search  Database - CINAHL Complete | 1,004 |
| S35 | S33 OR S34 | Expanders - Apply equivalent subjects Search modes - Proximity | Interface - EBSCOhost  Research Databases  Search Screen - Advanced  Search  Database - CINAHL Complete | 1,795,480 |
| S34 | "human health" | Expanders - Apply equivalent subjects Search modes - Proximity | Interface - EBSCOhost  Research Databases  Search Screen - Advanced  Search  Database - CINAHL Complete | 26,426 |
| S33 | S17 OR S18 OR S19 OR  S20 OR S21 OR S22 OR  S23 OR S24 OR S25 OR  S26 OR S27 OR S28 OR  S29 OR S30 OR S31 OR  S32 | Expanders - Apply equivalent subjects Search modes - Proximity | Interface - EBSCOhost  Research Databases  Search Screen - Advanced  Search  Database - CINAHL Complete | 1,772,808 |
| S32 | "human development" | Expanders - Apply equivalent subjects Search modes - Proximity | Interface - EBSCOhost  Research Databases  Search Screen - Advanced  Search  Database - CINAHL Complete | 5,501 |
| S31 | "child development" | Expanders - Apply equivalent subjects Search modes - Proximity | Interface - EBSCOhost  Research Databases  Search Screen - Advanced | 34,740 |

|  |  |  | Search  Database - CINAHL Complete |  |
| --- | --- | --- | --- | --- |
| S30 | "cognitive development" | Expanders - Apply equivalent subjects Search modes - Proximity | Interface - EBSCOhost  Research Databases  Search Screen - Advanced  Search  Database - CINAHL Complete | 3,040 |
| S29 | osteosarcoma* | Expanders - Apply equivalent subjects Search modes - Proximity | Interface - EBSCOhost  Research Databases  Search Screen - Advanced  Search  Database - CINAHL Complete | 5,142 |
| S28 | "health outcome*" | Expanders - Apply equivalent subjects Search modes - Proximity | Interface - EBSCOhost  Research Databases  Search Screen - Advanced  Search  Database - CINAHL Complete | 47,095 |
| S27 | "bladder cancer" | Expanders - Apply equivalent subjects Search modes - Proximity | Interface - EBSCOhost  Research Databases  Search Screen - Advanced  Search  Database - CINAHL Complete | 10,417 |
| S26 | "bladder syndrome" | Expanders - Apply equivalent subjects Search modes - Proximity | Interface - EBSCOhost  Research Databases  Search Screen - Advanced  Search  Database - CINAHL Complete | 392 |
| S25 | "down syndrome" | Expanders - Apply equivalent subjects Search modes - Proximity | Interface - EBSCOhost  Research Databases  Search Screen - Advanced Search  Database - CINAHL Complete | 9,598 |
| S24 | "renal calculi" | Expanders - Apply equivalent subjects Search modes - Proximity | Interface - EBSCOhost  Research Databases  Search Screen - Advanced  Search  Database - CINAHL Complete | 2,851 |
| S23 | "low intelligence quotient" | Expanders - Apply equivalent subjects Search modes - Proximity | Interface - EBSCOhost  Research Databases  Search Screen - Advanced  Search  Database - CINAHL Complete | 20 |

| S22 | "low IQ" | Expanders - Apply equivalent subjects Search modes - Proximity | Interface - EBSCOhost  Research Databases  Search Screen - Advanced  Search  Database - CINAHL Complete | 173 |
| --- | --- | --- | --- | --- |
| S21 | cancer* | Expanders - Apply equivalent subjects Search modes - Proximity | Interface - EBSCOhost  Research Databases  Search Screen - Advanced  Search  Database - CINAHL Complete | 576,008 |
| S20 | "hip fracture*" | Expanders - Apply equivalent subjects Search modes - Proximity | Interface - EBSCOhost  Research Databases  Search Screen - Advanced  Search  Database - CINAHL Complete | 16,335 |
| S19 | "non*dental human health outcome*" | Expanders - Apply equivalent subjects Search modes - Proximity | Interface - EBSCOhost  Research Databases  Search Screen - Advanced  Search  Database - CINAHL Complete | 0 |
| S18 | "non*dental outcome*" | Expanders - Apply equivalent subjects Search modes - Proximity | Interface - EBSCOhost  Research Databases  Search Screen - Advanced  Search  Database - CINAHL Complete | 1 |
| S17 | outcome* | Expanders - Apply equivalent subjects Search modes - Proximity | Interface - EBSCOhost  Research Databases  Search Screen - Advanced  Search  Database - CINAHL Complete | 1,258,994 |
| S16 | S11 OR S12 OR S13 OR  S14 OR S15 | Expanders - Apply equivalent subjects Search modes - Proximity | Interface - EBSCOhost  Research Databases  Search Screen - Advanced  Search  Database - CINAHL Complete | 7,888 |
| S15 | "community water fluor*" | Expanders - Apply equivalent subjects Search modes - Proximity | Interface - EBSCOhost  Research Databases  Search Screen - Advanced  Search  Database - CINAHL Complete | 163 |

| S14 | "community water fluor*" | Expanders - Apply equivalent subjects Search modes - Proximity | Interface - EBSCOhost  Research Databases  Search Screen - Advanced  Search  Database - CINAHL Complete | 163 |  |  |
| --- | --- | --- | --- | --- | --- | --- |
| S13 | fluorid* | Expanders - Apply equivalent subjects Search modes - Proximity | Interface - EBSCOhost  Research Databases  Search Screen - Advanced  Search  Database - CINAHL Complete | 7,888 |  |  |
| S12 | "water fluorid*" | Expanders - Apply equivalent subjects Search modes - Proximity | Interface - EBSCOhost  Research Databases  Search Screen - Advanced  Search  Database - CINAHL Complete | 598 |  |  |
| S11 | fluoride* | Expanders - Apply equivalent subjects Search modes - Proximity | Interface - EBSCOhost  Research Databases  Search Screen - Advanced  Search  Database - CINAHL Complete | 6,864 |  |  |
| S10 | S1 OR S2 OR S3 OR S4  OR S5 OR S6 OR S7 OR  S8 OR S9 | Expanders - Apply equivalent subjects Search modes - Proximity | Interface - EBSCOhost  Research Databases  Search Screen - Advanced  Search  Database - CINAHL Complete | 4,563,108 |  |  |
| S9 | adult | Expanders - Apply equivalent subjects Search modes - Proximity | Interface - EBSCOhost  Research Databases  Search Screen - Advanced  Search  Database - CINAHL Complete | 1,657,729 |  |  |
| S8 | Child* OR Adolesc* OR  Infant* OR Bab* OR Youth* OR “Young people” OR “Young person*” OR Teenage* OR parent* OR famil* | Expanders - Apply equivalent subjects Search modes - Proximity | Interface - EBSCOhost  Research Databases  Search Screen - Advanced  Search  Database - CINAHL Complete | 1,814,923 |  |  |
| S7 | child* | Expanders - Apply equivalent subjects Search modes - Proximity | Interface - EBSCOhost  Research Databases  Search Screen - Advanced  Search  Database - CINAHL Complete | 970,933 |  |  |
| S6 | adult* | Expanders - Apply equivalent subjects | Interface - EBSCOhost  Research Databases | 1,669,133 |  |  |
| Search modes - Proximity Search Screen - Advanced  Search  Database - CINAHL Complete  S5 person* Expanders - Apply Interface - EBSCOhost 814,521  equivalent subjects Research Databases  Search modes - Proximity Search Screen - Advanced Search  Database - CINAHL Complete  S4 peopl* Expanders - Apply Interface - EBSCOhost 335,719  equivalent subjects Research Databases  Search modes - Proximity Search Screen - Advanced  Search  Database - CINAHL Complete  S3 "human bein*" Expanders - Apply Interface - EBSCOhost 4,809  equivalent subjects Research Databases  Search modes - Proximity Search Screen - Advanced  Search  Database - CINAHL Complete  S2 human* Expanders - Apply Interface - EBSCOhost 3,078,825  equivalent subjects Research Databases  Search modes - Proximity Search Screen - Advanced  Search  Database - CINAHL Complete  S1 human* Expanders - Apply Interface - EBSCOhost 3,078,825  equivalent subjects Research Databases  Search modes - Proximity Search Screen - Advanced Search  Database - CINAHL Complete | | | | | | |

**File B. Characteristics of included studies**

| **Study** | **Country** | **Design** | **Outcome assessed** | **Type of Participants** | **Number of participants** | **Setting** | **Water Fluoride concentration (exposed group)** | **Water Fluoride concentration (control group)** | **Summary of findings** |
| --- | --- | --- | --- | --- | --- | --- | --- | --- | --- |
| Amini 2011 | Other: Iran | Cross sectional study | The relationship between fluoride concentrations in groundwater resources and blood pressure (both systolic and diastolic) as well as hypertension prevalence. |  |  |  | 0.23 to 1.86 mg/l, with a mean of 0.53 mg/l |  | There were statistically significant positive correlations between the mean concentrations of fluoride in groundwater resources and hypertension prevalence in males (r=0.48, p=0.007), females (r=0.36, p=0.048), and overall (r=0.495, p=0.005). Significant positive correlations were also found between fluoride concentrations and mean systolic blood pressure in males (r=0.431, p=0.018) and a borderline correlation in females (r=0.352, p=0.057). No significant correlations were found between fluoride concentrations and mean diastolic blood pressure in both males and females. The study concludes that increased fluoride levels in groundwater are associated with higher hypertension prevalence and mean systolic blood pressure, particularly in males. |
| Aravind 2016 | India | Cross sectional study | Intelligence quotient (IQ) of the children | Children (aged 6-12) | Total: 288 children (96 from each village)  Low fluoride area: 49% males, 51% females Medium fluoride area: 48% males, 52% females High fluoride area: 49% males, 51% females | School | Low fluoride area: < 1.2 ppm Medium fluoride area: 1.2–2 ppm High fluoride area: > 2 ppm |  | The study found a significant inverse relationship between fluoride concentration in drinking water and IQ levels in children. Higher fluoride concentrations were associated with lower IQ levels. The study concluded that IQ levels were negatively correlated with fluoride levels in drinking water, and it is necessary to consider factors that might affect children's IQ and devise solutions to prevent the harmful effects of excessive fluoride intake. |
| Archer 2016 | United States | Case control study | Childhood and adolescent osteosarcoma | Children (aged 0-5); Children (aged 6-12); Adolescents (aged 13-17); Adults (aged 18+) | Osteosarcoma cases: 308 (57.8% male, 42.2% female) CNS tumor controls: 604 (52.3% male, 47.7%) Leukemia controls: 598 (59.0% male, 41%) | Community | 0.1 to 5.5 ppm (mean 1.3+ ppm) | 0.1 to 5.5 ppm (mean ≤0.6 ppm) | The study found no significant association between fluoride levels in public drinking water and childhood/adolescent osteosarcoma. The odds of childhood osteosarcoma did not increase with increased PWS fluoride levels. This finding is consistent with the majority of studies on this topic, indicating that optimally fluoridated drinking water does not increase the risk of osteosarcoma in children. |
| Arun 2022 | United States | Cross sectional study |  | Adolescents (aged 13-17); Adults (aged 18+) | 7147 and 6858 women with complete birth weight and water fluoride data, respectively. | Community | Overall sample mean = 0.50 ± 0.04; Non-Hispanic White (NHW) = 0.46 ± 0.04 Non-Hispanic Black (NHB) = 0.56 ± 0.03 Hispanic = 0.55 ± 0.05 Other = 0.48 ± 0.04  Note:  Water supply source: Community water (overall participants = 3552 (72.0)), Well/Rain cistern (overall participants =313 (8.0)); (Spring = 65 (1.0)); Do not drink tap water = 1412 (19.0) |  | Women with LBW infants were exposed to significantly higher levels of water fluoride compared to those with normal birth weight infants. The findings suggest a significant association between excess water fluoride exposure (>0.7 ppm) and LBW weight in Hispanic women, independent of established LBW risk factors. In logistic regression models, Hispanic women exposed to increased levels of water fluoride were 1.5 times more likely to give birth to an LBW infant and 3.5 more likely to give birth to a VLBW infant. Taken together, these findings can inform public health education strategies that highlight water fluoride as a potential risk factor during pregnancy in Hispanic women. |
| Ballantyne 2022 | United States | Cross sectional study | Blood pressure among children and adolescents aged 8 to 19 years | Children (aged 6-12); Adolescents (aged 13-17); Adults (aged 18+) | 3495 (1762 female and 1733 male) | Community | Q1 = 0.30 ± 0.03 mg/L; Q2 = 0.46 ± 0.04 mg/L; Q3 = 0.55 ± 0.03 mg/L; Q4 = 0.67 ± 0.04 mg/L |  | While most other characteristics showed similar proportions and mean values across quartiles of plasma fluoride, there were tendencies for higher triglyceride (Q4: 102.7 ± 4.3 vs. Q1: 94.2 ± 3.1 mg/dL), water fluoride (Q4: 0.67 ± 0.04 vs. Q1: 0.30 ± 0.03 mg/L) and cotinine levels (Q4: 12.7 ± 2.5 vs. Q1: 4.5 ± 1.0 ng/L) with higher fluoride levels. Fasting duration was slightly shorter in the highest compared to the lowest quartile (Q4: 362.1 ± 13.8 min vs. Q1: 399.2 ± 13.9). There was a statistically significant positive correlation between water fluoride content and plasma fluoride concentrations (Spearman’s r = 0.41, p < 0.01 However, multivariable linear regression models did not show significant differences in adjusted mean values of systolic and diastolic blood pressure across increasing quartiles of fluoride concentrations. Further markers of cardio-metabolic health were not associated with fluoride status, with the exception of a weak inverse association between plasma fluoride and HbA1c levels. Higher plasma fluoride may not be a risk factor for increased blood pressure or impaired cardio-metabolic health among children in the USA, a non-fluoride endemic country, with wide-spread water fluoridation. |
| Barberio 2017 | Canada | Cross sectional study | The diagnosis of a learning disability | Children (aged 0-5); Children (aged 6-12) | Cycle 3: 1101 children Males: 51.75% Females: 48.25% | Canadian Health Measures Survey, which included household interviews and physical health measurements taken at mobile examination clinics. | 0.36 mg/L | 0.23 mg/L | Reported learning disability diagnosis was not significantly associated with any measure of fluoride exposure (urinary fluoride, creatinine-adjusted urinary fluoride, specific gravity-adjusted urinary fluoride, or fluoride concentration of tap water) in unadjusted or adjusted models. Overall, the study concluded that there did not appear to be a robust association between fluoride exposure from water and reported diagnosis of a learning disability among Canadian children. |
| Barberio 2017 | Canada | Cross sectional study | Diagnosis of a thyroid condition Thyroid-stimulating hormone (TSH) levels Free thyroxine (T4) levels | Adults (aged 18+) |  | Community (data collected through in-home interviews followed by clinical exams conducted in mobile clinics) | Mean 0.22 mg/L (only for Cycle 3) |  | Fluoride concentration or tap water (mg/L) did not differ between individuals classified as having primary hypothyroidism (mean=0.36mg/L, SE=0.11mg/L, 95%CI 0.16 to 0.57mg/L) versus not (mean=0.22mg/L, SE=0.04mg/L, 95%CI 0.15 to 0.30mg/L).   The study found no evidence of a relationship between fluoride exposure from tap water and the diagnosis of a thyroid condition. There was no statistically significant association between fluoride concentration in tap water and abnormal (low or high) TSH levels relative to normal TSH levels. These findings suggest that, at the population level in Canada, fluoride exposure from water does not contribute to impaired thyroid functioning during a time where multiple sources of fluoride exposure, including community water fluoridation, exist. |
| Bhatia 2024 | United States | Cohort study | Bone densitometry outcomes in young adults | Adults (aged 18+) | 185 female, 145 male | Community | 0.8-1 ppm |  | In fully adjusted analyses, no statistically significant (p < 0.01) or suggestive (0.01 < p < 0.05) associations were found between period-specific or cumulative fluoride intake and bone measures for either sex, although there were suggestive positive relationships in unadjusted analyses. Longitudinal fluoride intakes had little association with bone measures at age 23. As there were no adverse effects from fluoride intake on bone health in young adults, results support the continued use of fluorides, particularly community water fluoridation is the most cost-effective method of dental caries prevention |
| Blakey 2014 | UK | Cross sectional study | Risk of primary bone cancer | Children (aged 0-5); Children (aged 6-12); Adolescents (aged 13-17); Adults (aged 18+) | The study analysed 2566 osteosarcoma cases (1493 males, 1073 females) and 1650 ES cases (988 males, 662 females) | Case data on osteosarcoma and Ewing sarcoma, diagnosed at ages 0–49 years in Great Britain (GB) (defined here as England, Scotland and Wales) during the period 1980–2005, were obtained from population-based cancer registries. | The means range from 0.00 to 1.26 ppm |  | There was no evidence of an association between osteosarcoma risk and fluoride in drinking water [relative risk (RR) per one part per million increase in the level of fluoride ¼ 1001; 90% confidence interval (CI) 0871, 1151] and similarly there was no association for Ewing sarcoma (RR ¼ 0929; 90% CI 0773, 1115). Therefore, findings from this study provide no evidence that higher levels of fluoride (whether natural or artificial) in drinking water in GB lead to greater risk of either osteosarcoma or Ewing sarcoma. |
| Broadbent 2015 | Other: New Zealand | Cohort study | IQ in childhood/adolescence and adulthood | Children (aged 6-12); Adolescents (aged 13-17); Adults (aged 18+) | 3-17 years of age = 992 38 years of age = 942 | Community | 0.7-1.0 ppm | 0.0-0.3 ppm | The study found no clear differences in IQ due to fluoride exposure. These findings held after adjusting for potential confounding variables, including sex, socioeconomic status, breastfeeding, and birth weight (as well as educational attainment for adult IQ outcomes). The study concluded that fluoride in the context of community water fluoridation programs is not neurotoxic and does not support the assertion that it causes IQ deficits. |
| Chachra 2010 | Canada | Case control study | A relationship between fluoride content and structural or  mechanical properties of bone | Adults (aged 18+) | Out of 53 participants from Toronto, 26 were male and 27 female. Out of 39 participants from Montreal. 15 were male and 24 female. | Hospital | Toronto = 1.030 ± 0.060 (range 0.192-2.264) | Montreal = 0.643 ± 0.035 (range 0.270-1.200) | A weak relationship among fluoride exposure, accumulated fluoride, and the physical characteristics of bone was observed. Analysis of the data suggests  that the variability in heterogenous urban populations may be too high for the effects, if any, of low-level fluoride administration on skeletal tissue to be discerned. While the effect of low-level fluoride accumulation over long periods of time cannot be ruled out, it appears that the contributors to bone health are too many and varied, and any possible effect of municipal fluoride ingestion is too small, for municipal water fluoridation to be a significant determinant of bone health within the general public. |
| Chen 2013 | China | Cross sectional study | The study assessed changes in water fluoride, urinary fluoride (UF), and bone metabolism indicators in children after supplying low fluoride public water. | Children (aged 6-12) | 550 participants provided urine and 526 blood and urine samples | School | Village A - before intervention = 5.51 mg/L; after intervention = 0.11 mg/L Village B - before intervention = 2.17 mg/L; after intervention = 0.09 mg/L Village C - before intervention = 3.99 mg/L; after intervention = 0.11 mg/L Village D - before intervention = 3.31 mg/L; after intervention = 0.10 mg/L | Village E - before intervention = 0.12 mg/L; after intervention = 0.11 mg/L | The intervention of supplying low fluoride public water was successful, as evidenced by the reduction of fluoride in water and urine. Urinary fluoride concentrations in children from the intervention villages were lower or comparable to those in the control village after 10 years of supplying new public water. Most bone metabolism indicators in children from the intervention villages were either lower or similar to those in the control village after the intervention. Calcitonin (CT) and osteocalcin (BGP) were identified as the most sensitive bone metabolism indicators related to urinary fluoride. Bone mineral density showed the most stable and lowest abnormal rates over time. |
| CunninghamJEA 2021 | Canada | Cross sectional study | The study assessed sleep outcomes, including sleep duration, frequency of sleep problems, and daytime sleepiness. | Adolescents (aged 13-17); Adults (aged 18+) | Urinary fluoride sample (n = 1303) - 669 (51.3%) female and 634 (48.7%) male Water fluoride sample (n = 1016) - 515 (50.7%) female and 501 (49.3%) male | Home | Fluoridated tap water, mean (SD) F concentration 0.52 (0.22) mg/L | Non-fluoridated tap water, mean (SD) F concentration 0.05 (0.15) mg/L | A 0.5 mg/L higher water fluoride level was associated with 34% higher relative risk of reporting sleeping less than the recommended duration for age [unweighted: RRR = 1.34, 95% CI: 1.03, 1.73; p = .026]; the relative risk was higher, though less precise, using survey-weighted data [RRR = 1.96, 95% CI: 0.99, 3.87; p = .05]. UFSG was not significantly associated with sleep duration. Water fluoride and UFSG concentration were not significantly associated with frequency of sleep problems or daytime sleepiness. Therefore, fluoride exposure may contribute to sleeping less than the recommended duration among older adolescents and adults in Canada. |
| Das 2016 | India | Cross sectional study | Intelligence Quotient (IQ) Body Mass Index (BMI) | Children (aged 6-12); Adolescents (aged 13-17) | Total number of participants: 149 children 66 boys and 83 girls | Community | 0.25 to 9.40 mg/L, with an average value of 2.11 mg/L (±SD 1.64) |  | Intelligence Quotient (IQ)  Negative Correlation: Higher fluoride exposure is linked to lower IQ levels (r = -0.343, P<0.01). IQ Distribution: 24.16% of children had low IQ, while 12.08% had outstanding IQ. Impact: Children with moderate dental fluorosis had the lowest average IQ (84.51).  Body Mass Index (BMI)  Negative Correlation: Higher fluoride exposure is associated with lower BMI (r = -0.083). |
| DenBesten 2022 | United States | Cross sectional study | Inflammation, indicated by blood cell markers | Children (aged 6-12); Adolescents (aged 13-17); Adults (aged 18+) | Total Participants: 3,491 Males: 46% Females: 54% | Community (The study was conducted using data from the National Health and Nutrition Examination Survey (NHANES)) | Mean 0.56 ppm (range 0.07 to 7.32 ppm) |  | The study found that plasma fluoride was significantly positively associated with water fluoride concentrations and total WBC count, segmented neutrophils, and monocytes. There was a negative association with red blood cell count. These findings suggest that higher plasma fluoride levels are associated with increased inflammation in children and adolescents in the United States. |
| Do 2023 | Australia | Cohort study | Emotional and behavioral development Executive functioning | Children (aged 0-5); Children (aged 6-12) | 2,682 children (47.4% male and 52.6% female) | Community | ≥0.7 mg/L | N/A | The study found no association between exposure to fluoridated water during the first 5 years of life and altered measures of child emotional and behavioral development and executive functioning. Children with 100% lifetime exposure to fluoridated water had similar SDQ and BRIEF scores compared to those with 0% exposure. Factors such as low household income, identifying as Indigenous, and having a neurodevelopmental diagnosis were associated with poorer SDQ and BRIEF scores. |
| Do 2024 | Australia | Cohort study | The main outcome assessed was cognitive neurodevelopment, specifically measured by the full-scale intelligence quotient (FSIQ) using the Wechsler Adult Intelligence Scale 4th edition (WAIS-IV). | Adolescents (aged 13-17); Adults (aged 18+) | Total number: 357 0% LEFW (n = 68) = 41.2% male, 58.8% female >0% to <100% LEFW (n = 83) = 53.8% male, 46.4% female 100% LEFW (n = 194) = 47.4% male, 52.6% female | Clinic | 100% Lifetime Exposure to Fluoridated Water (100%LEFW): The fluoride concentration in public water supplies for this group was coded as 1 for levels of >0.7 to 1.1 mgF/L. | 0% Lifetime Exposure to Fluoridated Water (0%LEFW): The fluoride concentration for this group was set to 0 mg/L, indicating no exposure to fluoridated water. | The study found slightly higher FSIQ scores among those exposed to fluoride compared to those not exposed. The adjusted β of 100%LEFW versus 0%LEFW was 1.07 (95% CI: −2.86, 5.01), and of having dental fluorosis versus no fluorosis was 0.28 (95% CI: −3.00, 3.57). The hypothesis of noninferiority tests found that FSIQ scores of those exposed and nonexposed to fluoride were equivalent. The study provided evidence that early childhood exposure to fluoride does not have negative effects on cognitive neurodevelopment. |
| Farmus 2021 | Canada | Cohort study | Intelligence quotient (IQ) | Children (aged 0-5) | Total participants: 596 mother-child pairs 51.1% female children (303) and 48.9% male children (293) | Community | 0.7 mg/L |  | The study found that the association between fluoride exposure and Performance IQ (PIQ) significantly differed across prenatal, infancy, and childhood exposure windows. The strongest association was observed during the prenatal window, with a decrease in PIQ by 2.36 points per standard deviation increase in fluoride exposure. Boys showed a stronger negative association with prenatal fluoride exposure, while girls showed a stronger association during infancy. Fluoride exposure was not significantly associated with Verbal IQ (VIQ) across any exposure window. |
| Fluegge 2016 | United States | Cross sectional study | Age-adjusted diabetes incidence Age-adjusted diabetes prevalence | Adults (aged 18+) |  | Community | Artificial fluoridation: 0.71 ppm in 2005 and 0.31 ppm in 2010 | Naturally present fluoride: 0.23 ppm in 2005 and 0.27 ppm in 2010. | A 1 mg increase in the county mean added fluoride significantly predicted a 0.23 per 1,000 person increase in age-adjusted diabetes incidence and a 0.17% increase in age-adjusted diabetes prevalence. Natural fluoride concentration was significantly protective against diabetes. For counties using fluorosilicic acid as the chemical additive, both diabetes incidence and prevalence were lower. The study adjusted for various county-level and time-varying factors, including per capita tap water consumption, poverty, year, population density, age-adjusted obesity, and physical inactivity. Sensitivity analyses confirmed the robustness of the effects for both types of fluoride. |
| Goodman 2022 | Canada | Cohort study | Fertility Birth weight Gestational age Preterm birth Small-for-gestational age (SGA) | Adults (aged 18+) | Total Participants: 2001 pregnant women were initially recruited.  Participants with Available Data: Urinary Fluoride Measures: 1566 women Water Fluoride Concentration: 1370 women Fluoride Intake: 1192 women | Community | Median 0.52 mg/L (IQR: 0.17–0.64 mg/L) |  | No significant associations were observed between maternal fluoride exposure (measured through urinary fluoride, water fluoride, and fluoride intake) and fertility, birth weight, gestational age, preterm birth, or SGA. |
| Green 2019 | Canada | Cohort study | Children's intelligence quotient (IQ) | Children (aged 0-5) | Total participants: 601 mother-child pairs 52% female children (264) and 48% male children (237) | Clinic | Mean water fluoride concentration was 0.59 mg/L | Mean water fluoride concentration was 0.13 mg/L | The study found that higher maternal fluoride exposure during pregnancy was associated with lower IQ scores in children aged 3 to 4 years. A 1 mg/L increase in maternal urinary fluoride (MUF) was associated with a 4.49-point lower IQ score in boys, but there was no statistically significant association with IQ scores in girls. A 1 mg higher daily intake of fluoride among pregnant women was associated with a 3.66-point lower IQ score in both boys and girls. |
| Guo 2023 | United States | Cross sectional study | Blood pressure (both systolic and diastolic) | Children (aged 6-12); Adolescents (aged 13-17); Adults (aged 18+) | Total number of participants: 3260 Males: 1653 (50.71%) Females: 1607 (49.29%) | Community | 0.50 (0.03) mg/L |  | The study found that higher fluoride exposure from drinking water is associated with lower systolic blood pressure (SBP) in children and adolescents. Decrease in SBP: Each 1-mg/L increase in water fluoride concentration led to a 0.473 mm Hg decrease in SBP (Girls: 0.423 mm Hg decrease in SBP; Adolescents: 0.623 mm Hg decrease in SBP; Non-Hispanic Whites: 0.694 mm Hg decrease in SBP). No significant effect on diastolic blood pressure (DBP). These findings suggest that fluoride in drinking water may lower SBP, with variations based on sex, age, and race/ethnicity. |
| Hall 2023 | Canada | Cohort study | Hypothyroidism in pregnant women Child Full-Scale IQ (FSIQ) at ages 3 to 4 years | Children (aged 0-5); Adults (aged 18+) | Total number of participants: 1508 women Children: 52.2% male, 47.8% female | Clinic | 0.42 mg/L | 0.13 mg/L | The study found that fluoride in drinking water was associated with an increased risk of primary hypothyroidism in pregnant women. Specifically, a 0.5 mg/L increase in drinking water fluoride concentration was associated with a 1.65 times greater odds of primary hypothyroidism. Among women with normal thyroid peroxidase antibody (TPOAb) levels, the risk of primary hypothyroidism increased with increasing water fluoride. Additionally, children born to women with primary hypothyroidism had lower Full-Scale IQ scores compared to children of euthyroid women, especially among boys. |
| Hall 2024 | Canada | Cohort study | Thyroid hormones (i.e., TSH, FT4, and TT4) and anti­ bodies (i.e., anti-thyroglobulin [Tg] and anti-thyroid peroxidase [TPO]) | Adults (aged 18+) | 1876 (100% female) | Clinic | First quartile: 0.04-0.13 mg/L Second quartile: 0.13-0.52 mg/L Third quartile: 0.52-0.62 mg/L |  | Water fluoride concen­tration showed a U-shaped association with maternal FT4, whereby women with water fluoride concentrations in the second (0.13–0.52 mg/L) and third (0.52–0.62 mg/L) quartiles had significantly lower FT4 compared to those with levels in the first quartile (0.04–0.13 mg/L). Adjustment for maternal iodine status did not change the results. |
| Helte 2021 | Other: Sweden | Cohort study | Bone mineral density (BMD) and fracture incidence | Adults (aged 18+) | 4,306 women with urine fluoride and creatinine measurements and bone scans | Clinic | 1.0 mg/L | Categorized into groups: 0, 0.3, and 0.5 mg/L | Baseline BMD was slightly higher among women in the highest vs. lowest tertiles of fluoride exposure. Fluoride exposures were positively associated with incident hip fractures, with multivariable-adjusted hazard ratios of 1.50 and 1.59 for the highest vs. lowest tertiles of urine fluoride and dietary fluoride, respectively. Associations with other fractures were less pronounced for urine fluoride and null for dietary fluoride. Restricting the analyses to women with consistent long-term drinking water exposures prior to baseline strengthened associations between fractures and urinary fluoride. The findings suggest that high consumption of drinking water with a fluoride concentration of approximately 1 mg/L may increase both BMD and skeletal fragility in older women. |
| Hosur 2012 | India | Cross sectional study | Thyroid hormone levels (FT3, FT4, and TSH) | Children (aged 6-12); Adolescents (aged 13-17); Adults (aged 18+) | Study Group: 65 subjects (27 male and 38 female) Control Group: 10 subjects (5 male and 5 female) | School | 0.5 ppm to 4 ppm | < 1 ppm | The study found that all subjects with dental fluorosis had serum levels of thyroid hormones (FT3, FT4, and TSH) within the normal range, except for one individual with elevated TSH levels. Statistical significance was found when comparing FT3 and TSH values with different Dean’s index groups. The study concluded that thyroid hormone levels were not significantly altered in subjects with dental fluorosis, suggesting that more detailed investigations are needed. |
| Kheradpisheh 2018 | Other: Iran | Case control study | T3, T4 and TSH hormones | Adults (aged 18+) | Case group: 38 male (19.2%); 160 female (80.8%) Control group: 88 male (41.3%); 125 female (58.7%) | Community | 0-0.29 mg/L: 59 (29.8%) 0.3-0.5 mg/L: 139 (70.2%) | 0-0.29 mg/L: 65 (30.5%) 0.3-0.5 mg/L: 148 (69.5%) | The average amount of TSH and T3 hormones based on the levels of fluoride in two concentration levels 0–0.29 and 0.3–0.5 (mg/L) was statistically significant (P=0.001 for controls and P=0.001 for cases). In multivariate regression logistic analysis, independent variable associated with Hypothyroidism were: gender (odds ratio: 2.5, CI 95%: 1.6–3.9), family history of thyroid disease (odds ratio: 2.7, CI 95%: 1.6–4.6), exercise (odds ratio: 5.34, CI 95%: 3.2–9), Diabetes (odds ratio: 3.7, CI 95%: 1.7–8), Hypertension (odds ratio: 3.2, CI 95%: 1.3–8.2), water consumption (odds ratio: 4, CI 95%: 1.2–14). It was found that fluoride has impacts on TSH, T3 hormones even in the standard concentration of less than 0.5mg/L. Application of standard household water purification devices was recommended for hypothyroidism |
| Kim 2020 | United States | Case control study | The risk of osteosarcoma | Children (aged 6-12); Adolescents (aged 13-17); Adults (aged 18+) | 645 (255 female, 390 male) | Hospital |  |  | The study found no statistically significant increased risk for osteosarcoma associated with living in a fluoridated community. The adjusted OR for osteosarcoma and ever having lived in a fluoridated area for non-bottled water drinkers was 0.51 (95% CI, 0.31 to 0.84; P = 0.008), indicating a protective effect. For bottled water drinkers, the adjusted OR was 1.86 (95% CI, 0.54 to 6.41; P = 0.326), showing no significant association. Overall, the findings demonstrated that community water fluoridation is not associated with an increased risk for osteosarcoma. |
| Koh 2022 | United States | Cross sectional study | Blood pressure (both systolic and diastolic) in children and adolescents | Children (aged 6-12); Adolescents (aged 13-17); Adults (aged 18+) | 3894 children and adolescents Gender Distribution: 48.8% male and 51.2% female among those with normal blood pressure; 63.6% male and 36.4% female among those with high blood pressure. | Community (National Health and Nutrition Examination Survey (NHANES)) | 0.01 to 7.32 mg/L; normal BP: 0.49; high BP: 0.51 |  | Higher concentrations of fluoride in water were associated with lower systolic blood pressure (SBP) among adolescents (12–18 years). No significant association was found between fluoride in plasma and blood pressure across all ages. The odds ratio of high blood pressure for an increase in water fluoride was not significant. The study suggests that fluoride alone cannot be responsible for blood pressure changes, as several biological metabolic processes may influence its physiological effects. |
| Krzeczkowski 2024 | Canada | Cohort study | Visual acuity, Infant heart rate variability (HRV) | Children (aged 0-5); Adults (aged 18+) | Visual acuity sub-sample: Mothers: 100% female (337 in total) Infants: 47.18% female, 52.82% male  HRV sub-sample: Mothers: 100% female (306 in total) Infants: 48.37% female, 51.62% male | Clinic | Median 0.20 (IQR: 0.13–0.56) mg/L Visual acuity sub-sample (mean (SD)): 0.37 (0.25) mg/L HRV sub-sample (mean (SD)): 0.36 (0.25) mg/L |  | After adjustment for confounding variables, water fluoride concentration was associated with poorer infant visual acuity (B = -1.51; 95 % CI: − 2.14,-0.88) and HRV as indicated by lower RMSSD (B = -1.60; 95 % CI: −2.74,-0.46) but not SDNN.  Findings that prenatal fluoride exposure may  adversely affect visual acuity and ANS functioning in infants highlights the gestational period as a critical period of susceptibility to fluoride. |
| Kumar 2018 | India | Cross sectional study | Thyroid hormone levels (T3, T4, TSH) | Children (aged 6-12); Adolescents (aged 13-17) | N/A | Clinic (department of dentistry) | Group A1: 1.5 - 5 ppm Group A2: 1.8 - 5.8 ppm | 0.94 - 1.08 ppm | Mean thyroid stimulating hormone (TSH), water fluoride levels, urine fluoride levels, and serum fluoride levels of subjects in the intervention group were significantly higher than those in the control group. Thyroid hormone level derangement was seen in 54% of subjects in the control group and 67.5% of subjects in the intervention group. A positive correlation exists between fluorosis and thyroid functional activity. |
| Kumar 2023 | India | Cross sectional study | Vitamin D deficiency Fasting blood glucose levels (BGLs) | Adults (aged 18+) | 303 diabetic patients (56.43% males and 43.56% females) | Hospital | > 1.5 mg/L | < 1.5 mg/L | There was a significant negative correlation between water fluoride levels and vitamin D levels (rs = -0.777, p < 0.001). There was a positive correlation between water fluoride levels and fasting BGLs (rs = 0.178, p < 0.05). Participants from fluoride-endemic areas (F > 1.5 mg/L) had higher odds of severe vitamin D deficiency (odds ratio: 5.07, 95% CI: 1.9–13.2, p = 0.0009). The study concluded that vitamin D deficiency and fasting BGLs are significantly associated with water fluoride levels. The findings suggest that fluoride toxicity contributes to poor glycemic control and vitamin D deficiency. |
| Lee 2020 | Other: South Korea | Other: Ecological study | Hip fracture, osteoporosis, and bone cancer | Children (aged 0-5); Children (aged 6-12); Adolescents (aged 13-17); Adults (aged 18+) | CWF: 4,406,021 (male: 49,9%; female: 50.1%) Non-CWF: 2,270,959 (male: 49.6%; female: 50.4%) | Community |  |  | Comparing the CWF area with the non-CWF area, there was no clear evidence that exposure to CWF increased health risks at the town level in Cheongju since CWF was terminated after 2004. The posterior relative risks (RR) of hip fracture was 0.95 (95% confidence intervals 0.87, 1.05) and osteoporosis was 0.94 (0.87, 1.02). The RR in bone cancer was a little high because the sample size very small compared to the other bone diseases (RR = 1.20 (0.89, 1.61)). The relative risk of selected bone diseases (hip fractures, osteoporosis, and bone cancer) increased over time but did not increase in the CWF area compared to non-CWF areas. Therefore, it was concluded that the implementation of the water fluoridation program in CWF areas did not increase the risk of adverse health effects, typically bone diseases (hip fracture, osteoporosis, and bone cancer) compared to areas where it was never implemented. The results of this study provide evidence of benefits and no harmful effects on humans. |
| Lee 2024 | Other: South Korea | Cohort study | Neurodevelopmental status and incidence of febrile convulsion, epilepsy, mental retardation, attention deficit hyperactivity disorder (ADHD), anxiety disorder, depressive disorder, diabetes mellitus, hypothyroidism, congenital hypothyroidism, bone fractures, bone tumors, hepatic failure, chronic hepatitis, acute kidney failure, chronic kidney disease, and renal stones. | Children (aged 6-12) | Intervention: 29,991 (51.5% male, 48.5% female) Control: 22,881 (51.8% male, 48.2% female) | Community | 0.8±0.2 mg/L |  | Children in the fluoride-exposed group had a decreased risk of bone fractures [hazard ratio  (95% confidence interval, CI), 0.89 (0.82–0.93)] and increased risk of hepatic failures [1.85, (1.14–2.98)] compared to those in the unexposed group. Additionally, the risk ratio of abnormal neurodevelopmental  screening outcomes increased by 9%, but this was statistically uncertain (95% CI, 0.95–1.26). Therefore, fluoridated tap water was associated with an increased risk of hepatic failure but a decreased risk of bone fractures in children. The association between fluoridated tap water and neurodevelopmental screening outcomes at 6 years remains unclear, highlighting the need for further studies to clarify this association |
| Levy 2012 | United States | Other: Ecological study | The incidence of osteosarcoma | Children (aged 0-5); Children (aged 6-12); Adolescents (aged 13-17); Adults (aged 18+) | N/A (based on states) | Community | 0.7–1.2 mg/L | lower or equal to 1.2 mg/L | The study found no significant differences in osteosarcoma incidence rates between high and low CWF states for any age group or sex. The higher incidence rates among 15-19 year old males compared to females were not associated with the state fluoridation status. The study concluded that community water fluoridation has no influence on osteosarcoma incidence rates during childhood and adolescence. |
| Levy 2014 | United States | Cohort study | Bone measures, including bone mineral content (BMC) and bone mineral density (BMD) at various sites (whole body, lumbar spine, and hip). | Adolescents (aged 13-17) | Total participants: 415 with bone scans at age 15 years. Gender distribution: 183 females and 175 males. | Community | 0.8-1 ppm |  | The study found no significant relationships between daily fluoride intake and adolescents' bone measures in adjusted models. The findings suggest that fluoride exposures at typical levels for most US adolescents in fluoridated areas do not have significant effects on bone mineral measures. |
| Lindsay 2023 | United States | Cross sectional study | The study assessed the rate of pediatric fractures, specifically:  Supracondylar humerus fracture (SCHF) Both bone forearm fracture (BBFFx) Femur fracture (FFx) | Children (aged 0-5); Children (aged 6-12) | Total Participants: 106,423 Supracondylar Humerus Fracture (SCHF): 40,197 (47.5% female) Both Bone Forearm Fracture (BBFFx): 61,041 (40.6% female) Femur Fracture (FFx): 5,185 (32.2% female) | National insurance database (PearlDiver) | 0.39 to 0.7 mg/L |  | The study found positive correlations between the percentage of state water fluoridation and fracture rates for both bone forearm fracture (BBFFx) and femur fracture (FFx). Increased fracture rates were found in states with the highest quartiles of water fluoridation and fluoride levels for supracondylar humerus fracture (SCHF) and BBFFx. The findings suggest that higher levels of water fluoridation are associated with higher rates of certain pediatric fractures. |
| Liu 2019 | China | Cross sectional study | Anthropometric measurements (height, weight, and BMI z-scores) | Children (aged 6-12); Adolescents (aged 13-17) | Males: 1,242 (51.1%) Females: 1,188 (48.9%) | Community | 0.83 mg/L (95% CI: 0.81, 0.86) |  | The study found that low-to-moderate fluoride exposure is associated with increased BMI z-scores and a higher prevalence of overweight/obesity in children. The associations were stronger in girls than in boys, and children of fathers with lower education levels were more vulnerable to fluoride |
| Malin 2015 | United States | Cross sectional study | The prevalence of Attention-Deficit Hyperactivity Disorder (ADHD) among children and adolescents | Children (aged 0-5); Children (aged 6-12); Adolescents (aged 13-17) | Three subsamples of children aged 4-17 living in the U.S. were used to assess ADHD prevalence per state in: 2003: 79,264 children 2007: 73,123 children 2011: 76,015 children | Community | The study focused on the prevalence of artificial water fluoridation, which was recommended at 0.7 – 1.2 mg/L by the CDC's Department of Health and Human Services |  | State prevalence of artificial water fluoridation in 1992 significantly positively predicted state prevalence of ADHD in 2003, 2007 and 2011, even after controlling for socioeconomic status. A multivariate regression analysis showed that after socioeconomic status was controlled each 1% increase in artificial fluoridation prevalence in 1992 was associated with approximately 67,000 to 131,000 additional ADHD diagnoses from 2003 to 2011. Overall state water fluoridation prevalence (not distinguishing between fluoridation types) was also significantly positively correlated with state prevalence of ADHD for all but one year examined. |
| Malin 2019 | United States | Cross sectional study | Sleep patterns and daytime sleepiness among older adolescents | Adolescents (aged 13-17); Adults (aged 18+) | Total participants: 512 Males: 49.08% Females: 50.92% | Community (National Health and Nutrition Examination Survey (NHANES)) | Median concentration was 0.27 mg/L |  | Sleep Apnea: Higher water fluoride concentrations were associated with 1.97 times higher odds of reporting symptoms suggestive of sleep apnea (e.g., snorting, gasping, or stopping breathing while asleep) at least once per week. Bedtime and Wake Time: Each interquartile range (IQR) increase in water fluoride concentration was associated with a 24-minute later bedtime and a 26-minute later wake time. Daytime Sleepiness: There was some indication that higher water fluoride concentrations were associated with more frequent daytime sleepiness, although these associations were borderline statistically significant after correction for multiple comparisons. Snoring: Among males, higher water fluoride concentrations were associated with a 38% reduction in the odds of reporting snoring. The study suggests that fluoride exposure from water may contribute to changes in sleep cycle regulation and sleep behaviors among older adolescents. |
| Malin 2019 | United States | Cross sectional study | Kidney parameters included estimated glomerular filtration rate (calculated by the original Schwartz formula), serum uric acid, and the urinary albumin to creatinine ratio. Liver parameters were assessed in serum and included alanine aminotransferase, aspartate aminotransferase, alkaline phosphatase, blood urea nitrogen, gamma-glutamyl transferase, and albumin | Adolescents (aged 13-17); Adults (aged 18+) | Overall: 1985 (52.7% male, 47.3% female) Water sub-sample: 1742 (53.7% male, 46.3% female) | Community | Median 0.48 mg/L |  | A 1 mg/L increase in water fluoride was associated with a 0.93 mg/dL lower blood urea nitrogen concentration (95% CI: −1.44, −0.42; p = 0.007). s: Fluoride exposure may contribute to complex changes in kidney and liver related parameters among U.S. adolescents. As the study is cross-sectional, reverse causality cannot be ruled out; therefore, altered kidney and/or liver function may impact bodily fluoride absorption and metabolic processes |
| Manjunathappa 2023 | India | Cross sectional study | Serum alkaline phosphatase (ALP) levels Serum phosphate levels | Adults (aged 18+) | 180 - 100% female | Hospital | 2.65 ±1.29 ppm | 0.50 ±0.28 ppm | The fluoride concentration in drinking water was significantly positively correlated with the fluoride levels in urine and blood serum. Significant differences were observed in the ALP levels between the two groups in both maternal serum and cord blood, with lower ALP levels in the high-fluoride group. The phosphate levels in maternal serum were significantly higher in the high-fluoride group. Both simple and multivariate regression analyses revealed that the fluoride content in drinking water was significantly associated with the ALP level in cord blood and the phosphate level in maternal serum. |
| Meng 2023 | China | Cross sectional study | Osteoarthritis | Adults (aged 18+) | Case: 345 (36.2% male, 62.8% female) Control: 783 (30% male, 70% female) | Community | 0.44 (0.30, 1.17) mg/L | 0.34 (0.23, 0.76) mg/L | Logistic regression analysis showed that an increased fluoride exposure was a risk factor for KOA (WF: OR=1.318, 95% CI 1.162–1.495, p<0.001). After adjusting for covariates, the risk of KOA in the 4th quartile (Q) of WF was twice that of the 1st Q (OR=2.079, 95% CI 1.448–2.986, p<0.001). The population aged<60 exposed to the 4th Q of WF (or UF) had a higher risk than the population exposed to the 1st Q of WF (ORWF=1.958, 95% CI 1.249–3.070, p=0.003). Excessive fluoride dose in drinking water could increase the risk of KOA. Especially, the population with aged<60, male and obesity more likely to having KOA when they exposed to same higher fluoride. |
| Näsman 2013 | Other: Sweden | Cohort study | Risk of hip fracture | Adults (aged 18+) | Male: 251,721 Female: 221,565 Total: 473,277 | Community (nationwide registers in Sweden, including the Swedish National In-Patient Register (IPR), the Swedish Cause of Death Register, and the Register of Population and Population Changes) | Very low: < 0.3 mg/L Low: 0.3 - 0.69 mg/L Medium: 0.7 - 1.49 mg/L High: ≥ 1.5 mg/L |  | The study found no association between chronic fluoride exposure from drinking water and the overall risk of hip fracture. The risk estimates did not change in analyses restricted to low-trauma osteoporotic hip fractures. There was no notable difference in adjusted relative risk estimates for hip fractures stratified by exposure group. The study suggested a potential protective effect of fluoride among individuals younger than 80 years, but this effect was not observed in the oldest age group. The study concluded that long-term fluoride exposure from drinking water does not seem to have any important effects on the risk of hip fracture within the investigated exposure range. |
| Peckham 2015 | UK | Cross sectional study | Hypothyroidism prevalence |  | N/A (based on the overall population) | Community | ≤ 0.3 mg/L >0.3 ≤ 0.7 mg/L > 0.7 mg/L |  | Study found that higher levels of fluoride in drinking water provide a useful contribution for predicting prevalence of hypothyroidism. Practices located in the West Midlands (a wholly fluoridated area) are nearly twice as likely to report high hypothyroidism prevalence in comparison to Greater Manchester (non-fluoridated area) |
| Saxena 2012 | India | Cross sectional study | A questionnaire was used to collect information on the  children’s personal characteristics, residential history, medical history, educational level of the head of the family, and socioeconomic status of the family. | Children (aged 6-12) | 170 in total <1.5 ppm = 54% male, 46% female 1.5-3.0 ppm = 51.3% male, 48.7% female 3.1-4.5 ppm = 48.8% male, 51.2% female >4.5 ppm = 52.6% male, 47.4% female | School | > 1.5 ppm (1.5-3.0 ppm; 3.1-4.5 ppm; >4.5 ppm) | < 1.5 ppm | Differences in participant’s sociodemographic characteristics, urinary iodine, urinary lead, and urinary arsenic levels were statistically not significant (P>0.05). However, a statistically significant difference was observed in the urinary fluoride levels (P 0.000).  Reduction in intelligence was observed with an increased water fluoride level (P 0.000). The urinary fluoride level was a significant predictor for intelligence (P 0.000). Children in endemic areas of fluorosis are at risk for impaired development of intelligence. |
| Sebastian 2015 | India | Cross sectional study | Intelligence Quotient (IQ) of the children | Children (aged 6-12) | 405 | School | Nerale: 2.0 mg F/l (high fluoride) Belavadi: 1.2 mg F/l (normal fluoride) Naganahally: 0.40 mg F/l (low fluoride) |  | The study found a significant relationship between water fluoride levels and the IQ of school children. Children in the high fluoride village (Nerale) had a larger proportion of IQ scores below 90 (below average IQ) compared to those in the normal and low fluoride villages. The study concluded that higher water fluoride levels were associated with impaired development of intelligence in children. Age, gender, parental education level, and family income did not show a significant association with IQ scores. |
| Seraj 2012 | Other: Iran | Cross sectional study | Intelligence quotient (IQ) of the children. | Children (aged 6-12) | Males: 142 Females: 151 Total: 293 | Community (five rural villages in Makoo, Iran) | Medium Fluoride: 3.1±0.9 ppm High Fluoride: 5.2±1.1 ppm Normal Fluoride: 0.8±0.3 ppm |  | The study found that the mean IQ scores decreased with increasing fluoride levels in drinking water. The mean IQ scores were:  Normal Fluoride Group: 97.77±18.91 Medium Fluoride Group: 89.03±12.99 High Fluoride Group: 88.58±16.01 Children residing in areas with higher than normal water fluoride levels demonstrated more impaired development of intelligence. The study concluded that children's intelligence might be affected by high water fluoride levels. |
| Shaik 2019 | India | Cross sectional study | Thyroid function, specifically serum levels of T3, T4, and TSH. | Children (aged 6-12); Adolescents (aged 13-17) | The study included 293 participants:  Group I (0.01-0.6 ppm fluoride): 98 children (39 boys and 59 girls) Group II (0.7-1.2 ppm fluoride): 103 children (42 boys and 61 girls) Group III (1.3-1.8 ppm fluoride): 92 children (55 boys and 37 girls) | Community | The fluoride concentrations in the water supplies of the villages were:  Group I: 0.01-0.6 ppm (mean 0.22 ppm) Group II: 0.7-1.2 ppm (mean 0.89 ppm) Group III: 1.3-1.8 ppm (mean 1.44 ppm) |  | The study found that long-term intake of fluoridated drinking water (0.02-1.4 ppm) did not significantly affect thyroid function in children with normal nutritional status and optimal iodine intake. The prevalence of deranged thyroid function (TSH and T4 levels) was observed but showed no significant association with the fluoride levels in drinking water. The study concluded that within the fluoride range studied, there was no significant impact on thyroid function. |
| Singh 2014 | India | Cross sectional study | Thyroid hormone levels (FT3, FT4) and thyroid-stimulating hormone (TSH) levels | Children (aged 6-12); Adolescents (aged 13-17) | Overall 70 Group 1 - 60 participants equally divided into two subgroups: Group 1A (children with dental fluorosis) and Group 1B (children without dental fluorosis). Group 2 included 10 children a non-endemic area | School | 1.6–5.1 ppm | 0.98–1 ppm | The study found that children in the endemic fluorosis area had significantly higher levels of fluoride in their serum and urine compared to the control group. There was a significant relationship between water fluoride levels and serum/urine fluoride concentrations. Additionally, children in the endemic area showed altered levels of thyroid hormones (FT3, FT4) and TSH, indicating thyroid dysfunction. |
| Till 2020 | Canada | Cohort study | Intellectual ability (IQ scores) in children | Children (aged 0-5) | Total Participants: 398 mother-child pairs Breastfed Group (BF): 200 Formula-fed Group (FF): 198  Breastfed Group (BF): Females: 51% Males: 49%  Formula-fed Group (FF): Females: 54% Males: 46% | Community | 0.59 mg/L | 0.13 mg/L | An increase of 0.5 mg/L in water fluoride concentration was associated with a 9.3-point decrement in Performance IQ among formula-fed children and a 6.2-point decrement among breastfed children. The association between water fluoride concentration and Performance IQ remained significant after controlling for fetal fluoride exposure. The study concluded that exposure to increasing levels of fluoride in tap water was associated with diminished non-verbal intellectual abilities, with a more pronounced effect among formula-fed children. |
| Wang 2020 | China | Cross sectional study | Thyroid function (measured through basal thyroid hormones and thyroid-stimulating hormone levels). Intelligence (measured through IQ scores). | Children (aged 6-12); Adolescents (aged 13-17) | Male: 292 (51.1%) Female: 279 (48.9%) | Community ( rural areas of Tianjin City, China) | Water fluoride concentration ranged from 0.20 mg/L to 3.9 mg/L, with a mean value of 1.39 ± 1.01 mg/L |  | Thyroid Function: Every 1 mg/L increment of water fluoride was associated with a 0.13 uIU/mL increase in TSH. Urinary fluoride was associated with decreases in TT4 and FT4 and an increase in TSH. Intelligence: Fluoride exposure was inversely related to IQ scores. Higher TT3 and FT3 were related to increased odds of children having high normal intelligence. There was a significant modification effect by TSH on the association between urinary fluoride and IQ scores. |
| Wang 2021 | China | Cross sectional study | Cholinergic system and IQ | Children (aged 6-12) | Total: 709 Male: 53.74%  Female: 46.26% | Community | 0.20 mg/L to 3.90 mg/L, with a mean value of 1.20 ± 0.95 mg/L |  | Children in the fourth quartile of water fluoride had a 20% increased risk of having an IQ < 120 compared to those in the first quartile. The risk of having both IQ < 120 and increased AChE levels was 58% and 62% in the third and fourth quartiles for water fluoride. Cholinergic System and Fluoride Exposure:  Water Fluoride: Positively associated with AChE levels Negatively associated with ChAT and ACh levels  The risk of either having non-high intelligence increased by 22% for children in the fourth quartile of AChE compared to the first quartile. The risk of having both non-high intelligence and increased AChE levels was 1.27, 1.37, and 1.44 times higher in the second, third, and fourth quartiles of AChE, respectively. AChE mediated 15.7% of the relationship between water fluoride and having an IQ < 120. For both conditions to exist, the mediation proportion was 6.7% for water fluoride.  Low-to-moderate fluoride exposure was associated with dysfunction of the cholinergic system in children. Acetylcholinesterase (AChE) may partly mediate the lower probability of having superior and above intelligence. |
| Wei 2021 | United States | Cross sectional study | Hyperuricemia | Children (aged 6-12); Adolescents (aged 13-17); Adults (aged 18+) | Total number = 1933 52.08% male, 47.92% female | Community (National Health and Nutrition Examination Survey (NHANES)) | Median value of 0.50 mg/L (range: 0.01–7.32 mg/L) |  | The study found a significant association between higher water fluoride concentrations and the prevalence of hyperuricemia among adolescents. There was a dose-dependent increase in the prevalence of hyperuricemia with increasing quartiles of water fluoride concentrations. Adolescents in the highest quartile of water fluoride (≥0.73 mg/L) had significantly increased odds of hyperuricemia compared to those in the lowest quartile. Adolescents who consumed water with fluoride concentrations in the highest quartile had a 1.75-fold increased odds of hyperuricemia (OR: 1.75; 95% CI: 1.04, 2.93). A positive relationship was observed between water fluoride concentrations and plasma fluoride concentrations, indicating that water fluoride is a significant source of fluoride exposure. These findings suggest that higher fluoride concentrations in drinking water are associated with an increased prevalence of hyperuricemia in adolescents. |
| Young 2015 | UK | Cross sectional study | Hip fracture Renal calculi (kidney stones) All-cause mortality Down syndrome Bladder cancer Osteosarcoma Overall cancer incidence | Children (aged 0-5); Children (aged 6-12); Adolescents (aged 13-17); Adults (aged 18+) | N/A (based on small Geographical Information Systems) | Community | 1 - 1.5 ppm | < 1 ppm | There was no strong evidence of an association between fluoridation and hip fracture, Down syndrome, all-cancer, all-cause mortality, or osteosarcoma. Fluoridation was negatively associated with the incidence of renal stones and bladder cancer. The study concludes that fluoridation is a safe and effective public health measure to reduce dental decay, with no evidence of detrimental health effects associated with fluoridation. |
| Yousefi 2018 | Other: Iran | Cross sectional study | The prevalence of hypertension, body mass index (BMI), waist circumference, and waist-to-hip ratio (WHR). | Adults (aged 18+) | High F area: 85 males, 105 females Low F area: 73 males, 83 females | Community (West Azerbaijan Province, Iran) | 0.68 to 10.30 mg/L |  | The study found a significant positive correlation between high fluoride levels in drinking water and increased prevalence of hypertension. The odds ratio of hypertension in residents with high fluoride levels was 2.3 times higher than those with lower fluoride levels. There were no significant correlations between fluoride levels and BMI, hip circumference, or WHR. |
| Yu 2018 | China | Cross sectional study | Intelligence quotient (IQ). | Children (aged 6-12); Adolescents (aged 13-17) | Water fluoride ≤1.0 mg/L: 849 (51.9%) male, 787 (48.1%) female Water fluoride >1 mg/L: 667 (53.4%) male, 583 (46.6%) female | Community (rural areas of Tianjin City, China) | Water fluoride ≤1.0 mg/L: 0.50 ± 0.27 mg/L Water fluoride >1.0 mg/L: 2.00 ± 0.75 mg/L |  | Average IQ was slightly higher in the normal-fluoride group (107.4) compared to the high-fluoride group (106.4). Significant IQ score reduction at water fluoride levels between 3.40 mg/L and 3.90 mg/L (4.29 points decrease per 0.5 mg/L increment). 47% lower probability of developing excellent intelligence (IQ ≥ 130) in the high-fluoride group. These findings indicate that moderately excessive fluoride exposure from drinking water is associated with reduced IQ scores and a lower probability of developing excellent intelligence in children. |
| Zhang 2019 | United States | Cross sectional study | Preterm birth (birth before 37 weeks of gestation) | Adults (aged 18+) | The study included 2,908 women in the "Community Water Fluoridation Only" group | Community | Not reported | Not reported | The prevalence of preterm birth among women with a singleton live birth was 8.5% in Massachusetts during the study period. 30.1% of women reported living in a community with water fluoridation but did not have their teeth cleaned during pregnancy. The association between CWF alone and preterm birth was not significant (adjusted risk ratio [aRR] = 0.81, 95% CI 0.63–1.05), compared to women without dental cleaning and CWF. These findings suggest that community water fluoridation alone was not significantly associated with a reduced prevalence of preterm birth. |
